# Effects of Fishmeal Replacement with Insect Meals on Growth Performance in Non-Fish Aquatic Animals: A Meta-Analysis

**DOI:** 10.3390/insects17070699

**Published:** 2026-07-06

**Authors:** Yao Lu, Yiyi Yu, Liefeng Li, Haojie Li, Shuyin Hu, Xingbang Qiu, Xiang Meng, Junjie Hu

**Affiliations:** 1 Key Laboratory of Conservation and Application in Biodiversity of South China, School of Life Sciences, Guangzhou University, Guangzhou 510006, China; luyao@e.gzhu.edu.cn (Y.L.); w34729511@163.com (Y.Y.); 32314100093@e.gzhu.edu.cn (L.L.); haojie.li@e.gzhu.edu.cn (H.L.); hushuyin@e.gzhu.edu.cn (S.H.); qwe135asdplm@163.com (X.Q.); 2 Guangdong Key Laboratory of Animal Conservation and Resource Utilization, Guangdong Public Laboratory of Wild Animal Conservation and Utilization, Institute of Zoology, Guangdong Academy of Sciences, Guangzhou 510260, China

**Keywords:** insect meal, fishmeal replacement, *Hermetia illucens*, *Tenebrio molitor*, crustacean, growth performance, aquafeed, meta-analysis

## Abstract

The aquafeed industry is seeking sustainable alternatives to fishmeal, and insect meals are promising candidates. However, quantitative syntheses of their effects on non-fish aquatic animals like shrimp, crabs, turtles, and frogs are limited. This meta-analysis evaluated five insect meals—black soldier fly, Coleoptera, housefly, silkworm, and Orthoptera—on specific growth rate (SGR), weight gain rate (WGR), and feed conversion ratio (FCR). A total of 69 studies (2004–2025) were included. Hedges’ *g* was pooled using random-effects models, and meta-regressions examined dose–response relationships. Among all categories, only silkworm meal improved or maintained growth, showing no significant negative dose–response relationship. In contrast, black soldier fly and Coleoptera meals impaired growth, with a negative linear dose–response. Subgroup analyses revealed species-specific responses; shrimp, particularly *Litopenaeus vannamei*, exhibited growth depression, whereas some crab species, including *Scylla paramamosain* and *Eriocheir sinensis*, showed tolerance or improved performance. Housefly and Orthoptera meals were evaluated in fewer studies, precluding firm conclusions. Thus, the efficacy of insect meal substitution is jointly determined by insect type and host physiology. Silkworm pupae meal is a favourable fishmeal alternative, while black soldier fly and Coleoptera meals require conservative inclusion limits, especially for shrimp.

## Simple Summary

Fishmeal is the main protein source in aquafeeds, but its supply is unsustainable. Insect meals are emerging as a promising alternative, yet their effects on shrimp, crabs, turtles, and frogs remain unclear, as existing research has focused primarily on fish. This study systematically integrated results from 69 published experiments to evaluate how five different insect meals—black soldier fly (*Hermetia illucens*), yellow mealworm, housefly, silkworm, and grasshoppers/crickets—affect growth and feed efficiency in these non-fish aquatic animals. The results showed that silkworm pupae meal consistently supported good growth, even at high inclusion levels. In contrast, black soldier fly and yellow mealworm meals tended to reduce growth as their inclusion increased, especially in shrimp. Crabs showed more varied responses, with some species tolerating these meals well. These findings provide practical guidance for the aquaculture industry on selecting insect-based protein sources and setting safe replacement levels to maintain animal performance. By supporting the sustainable use of insect meals in shrimp and crab farming, this work contributes to reducing reliance on fishmeal and promoting more environmentally responsible aquaculture.

## 1. Introduction

Global aquaculture represents one of the most rapidly expanding sectors of food production, contributing substantially to global food security and economic development, as reported by the Food and Agriculture Organization of the United Nations [1,2]. In 2022, global aquaculture production reached a record 94.4 million tonnes, with finfish accounting for the largest share [3]. Among non-fish aquatic animals, crustaceans, turtles, and frogs also contribute substantially to the sector’s economic value. In particular, crustaceans command market prices significantly higher than those of finfish [4], and their processing by-products are rich in high-value compounds such as chitin and chitosan, creating an additional value chain that is largely absent in fish [5]. The intensive culture of high-value crustaceans and other non-finfish species relies heavily on formulated feeds, in which fishmeal has long been the preferred protein source owing to its balanced amino acid profile, high palatability, and absence of anti-nutritional factors [6,7,8]. However, the sustainability of fishmeal supply is increasingly threatened by stagnating capture fisheries and rising demand, driving the search for alternative protein sources [3,9].

Among various alternative protein sources, insect meals have gained considerable attention as promising fishmeal substitutes in aquafeeds, driven by the rapid growth of the insect farming industry and its recognised environmental benefits [10]. Commonly evaluated species include black soldier fly (*Hermetia illucens*), yellow mealworm (*Tenebrio molitor*), housefly (*Musca domestica*), silkworm (*Bombyx mori*), and Orthoptera species (e.g., crickets and grasshoppers). These insect meals typically contain 40–63% crude protein and exhibit species-specific amino acid profiles that can be nutritionally comparable to fishmeal in some cases, although considerable variation exists among insect species [11,12]. Their production requires significantly less land, water, and feed than conventional livestock, thereby offering a substantially lower environmental footprint. Furthermore, insect farming enables the bioconversion of organic side-streams, aligning with circular economy principles [13,14]. Moreover, insect meals are rich in bioactive constituents, particularly chitin and antimicrobial peptides, which may confer additional functional benefits, including immunomodulation and improved gut health, in farmed aquatic animals [15]. The European Union authorised the use of processed animal protein derived from *H. illucens*, yellow mealworm, and housefly in aquafeeds in 2017 and subsequently added silkworm (*Bombyx mori*) in 2021 (Regulations EU 2017/893 and 2021/1925) [16,17].

Numerous feeding trials have evaluated the effects of insect meals on growth performance in non-fish aquatic animals, but the results are highly variable depending on host species, insect type, inclusion level, and processing method [6,18]. Although several narrative reviews have summarised these findings, the evidence remains fragmented and precludes robust quantitative estimation of effect sizes [11,12,19]. Existing meta-analyses examining insect meals in aquafeeds have focused predominantly on finfish species, and even those that included a limited number of crustacean studies did not analyse non-fish taxa as a distinct subgroup [20,21,22]. Consequently, the comparative efficacy of different insect categories across multiple non-fish hosts has not been systematically evaluated within a unified framework. Moreover, the majority of these fish-centric meta-analyses have examined only a single insect species (e.g., *H. illucens*), further limiting their utility for broader insect meal selection [23,24,25]. Consequently, a quantitative synthesis specifically targeting non-fish aquatic animals and systematically comparing multiple insect meal types remains lacking.

The present study therefore aimed to conduct a systematic review and meta-analysis quantifying the overall effects of five insect meal categories—*H. illucens*, Coleoptera (mainly *T. molitor*), *M. domestica*, *B. mori*, and Orthoptera—on specific growth rate (SGR), weight gain rate (WGR), and feed conversion ratio (FCR) in non-fish aquatic animals (shrimp, crabs, crayfish, turtles, and frogs). Subgroup analysis by host species and meta-regression of replacement level were performed to explore sources of heterogeneity. The findings are expected to provide species-specific recommendations for the practical use of insect meals in non-fish aquaculture.

## 2. Materials and Methods

### 2.1. Literature Search

**Figure 1:**
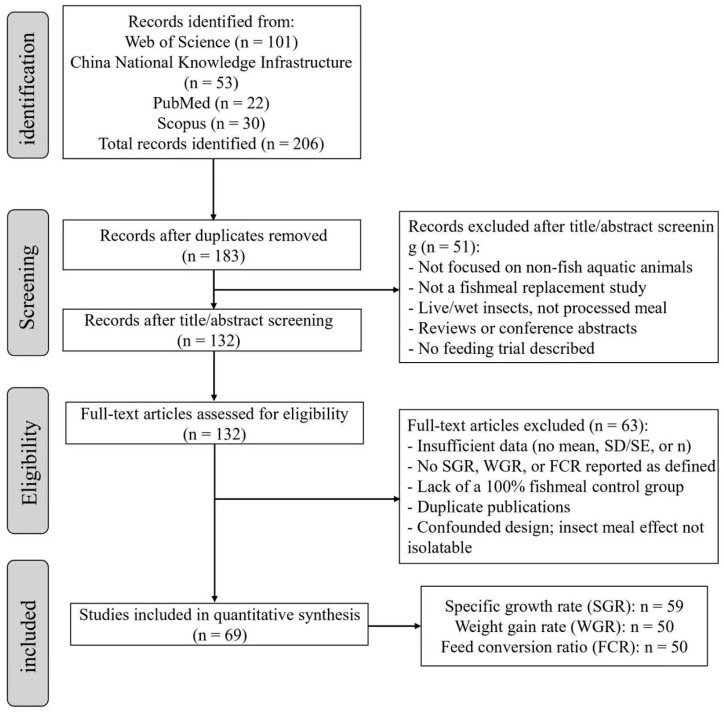
Preferred Reporting Items for Systematic Reviews and Meta-Analyses (PRISMA) flow chart for the eligible literature for the meta-analysis database.

This systematic review was conducted in accordance with the Preferred Reporting Items for Systematic Reviews and Meta-Analyses (PRISMA) 2020 guidelines as described by Page et al. [26]. A comprehensive literature search was performed across four electronic databases: Web of Science Core Collection (1965–2025), China National Knowledge Infrastructure (1994–2025), PubMed (1966–2025), and Scopus (1970–2025). The final search was conducted on 1 March 2025 (Figure 1). The following search string was applied in the Title, Abstract, and Keywords fields.

(“insect meal” OR “*Tenebrio molitor*” OR “Insecta” OR “*Hermetia illucens*” OR black soldier fly OR “*Musca domestica*” OR housefly OR “Imbrasia belina” OR worm OR “Chironomids” OR “*Gryllus bimaculatus*” OR cricket OR “*Bombyx mori*” OR silkworm OR “Oxya hyla hyla” OR grasshopper OR locust OR “Zonocerus variegatus” OR “Zophobas morio” OR “Cirina butyrospermi” OR “Gryllodes sigillatus” OR “Blatta lateralis” OR cockroach OR “Oxya fuscovittata” OR “*Acheta domesticus*” OR “Gryllus assimilis” OR “Termite”) AND (“fish meal” OR “fishmeal”) AND (crab OR shrimp OR turtle OR frog OR shellfish OR salamanders) AND (growth).

The studies were eligible for inclusion in the meta-analysis database if they met the following criteria: (i) includes insect meals as an alternative protein source for fishmeal in diets; (ii) focuses on non-fish aquatic animals; (iii) reports one or more of the following production performance parameters: specific growth rate (SGR), weight gain rate (WGR), or feed conversion ratio (FCR); (iv) provides sufficient information on investigated parameters, such as mean, sample size, and treatment error (standard deviation [SD], standard error [SE]). The studies that investigated mixed insect meals or a mixture of insect meals vs. other protein sources as a replacement for fishmeal were not eligible for the database.

### 2.2. Data Extraction and Assessment

The following data were extracted from each included study: first author, publication year, experimental subject (common and scientific names), insect meal type (common and scientific names), and fishmeal replacement level (%). For growth performance metrics, the sample size (n), mean, and measure of variability (standard deviation or standard error) were recorded for both the control and each treatment group. Studies with multiple treatment groups (e.g., different replacement levels) contributed multiple effect sizes, with each treatment group compared independently to the same control group. All extracted data were compiled in an Excel spreadsheet. Data were extracted independently by two reviewers and cross-checked by the supervisor.

The growth performance parameters in the included studies were calculated using the following standard equations:

Weight gain rate (WGR, %) = 100% × (Final weight − Initial weight)/Initial weight

Specific growth rate (SGR, %/day) = 100% × [ln (final weight) − ln (initial weight)]/days

Feed conversion ratio (FCR) = Feed intake/Weight gain

### 2.3. Data Analysis

Hedges’ *g* effect size was calculated to quantify the standardised mean differences between insect meal-containing and control diets [27]. For SGR and WGR, a positive Hedges’ *g* indicated improved growth performance relative to the control, whereas for FCR, a positive g indicated poorer feed efficiency (i.e., higher FCR values). A random-effects model (DerSimonian–Laird estimator) was applied to pool effect sizes, given the anticipated heterogeneity arising from variations in aquatic animal species, insect meal types, and experimental conditions.

To evaluate differences among insect meal types, Kruskal–Wallis tests were used to compare effect sizes for specific growth rate (SGR), weight gain rate (WGR), and feed conversion ratio (FCR), as these data did not follow a normal distribution. When significant differences were detected, Dunn’s post hoc tests with Bonferroni correction were applied for multiple comparisons.

Subgroup analyses were further conducted by aquatic animal species within major insect meal categories to explore potential sources of heterogeneity. Such subgroup analyses were performed only for insect meal categories with at least three host species, each contributing three or more independent effect sizes, to ensure statistical reliability of subgroup estimates. Additionally, random-effects meta-regression was performed to examine the relationship between effect size and fishmeal replacement level (%) as a continuous moderator.

### 2.4. Heterogeneity and Publication Bias

Heterogeneity among studies was assessed using Cochran’s *Q* test and quantified by the *I*^2^ statistic. An *I*^2^ value greater than 50% was considered indicative of substantial heterogeneity, whereas an *I*^2^ value of 50% or less indicated low heterogeneity [28].

Publication bias was assessed visually using funnel plots and statistically using Egger’s linear regression test [29]. For meta-analyses involving at least 10 studies, Egger’s test was performed, with *p* < 0.05 indicating potential publication bias. When significant publication bias was detected, the trim-and-fill method was applied to estimate the number of missing studies and compute adjusted effect sizes [30]. Sensitivity analysis was conducted by sequentially removing each study and recalculating the pooled effect size to evaluate the robustness of the results.

Effect size calculation (Hedges’ *g*) was performed using MetaWin 3.0 (https://www.metawinsoft.com, accessed on 1 March 2025). All other statistical analyses, including meta-regression, publication bias tests, and non-parametric comparisons (Kruskal–Wallis test with Dunn’s post hoc test), as well as graphical outputs (forest plots, funnel plots), were conducted using R version 4.3.1 (https://www.r-project.org, accessed on 1 March 2025; R Core Team, 2023). No generative AI was used in this study.

## 3. Results

### 3.1. Overview of Included Studies

**Figure 2:**
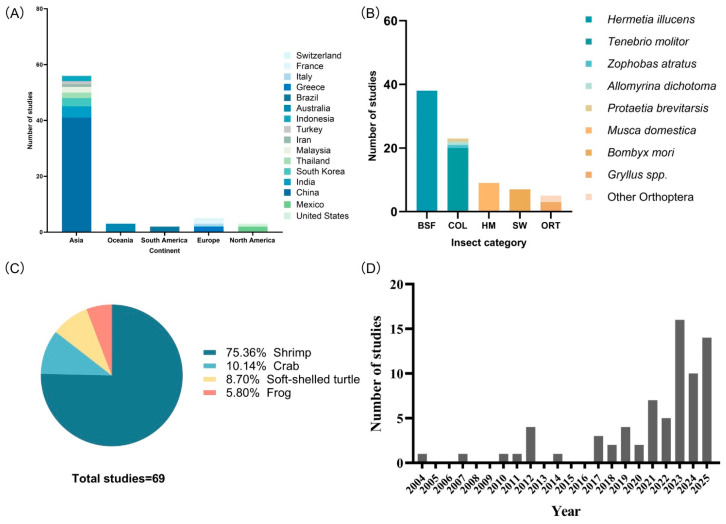
Overview of the dataset. (**A**) distribution of publications by continent and country, (**B**) distribution of publications by taxonomic group, (**C**) frequency of insect meal categories across studies, (**D**) cumulative number of publications by year. Insect species abbreviations: BSF, *Hermetia illucens* (black soldier fly); COL, Coleoptera (primarily *Tenebrio molitor*, yellow mealworm); HM, *Musca domestica* (housefly); SW, *Bombyx mori* (silkworm); ORT, Orthoptera (crickets/grasshoppers).

A total of 69 studies published between 2004 and 2025 were included in the meta-analysis database (Figure 1; Table S1). The studies spanned 17 countries, with China contributing the largest proportion (*n* = 40, 58.0%), followed by India (*n* = 4, 5.8%) and then Australia and South Korea (each *n* = 3, 4.3%) (Figure 2A). The dataset comprised shrimp, crab, soft-shelled turtle, and frog. Shrimp were the most studied group, accounting for 62.3% (*n* = 43) of all studies, with Pacific white shrimp (*Litopenaeus vannamei*) being the predominant species (*n* = 34) (Figure 2B). Five insect meal categories were identified, with some studies evaluating multiple categories: black soldier fly (*Hermetia illucens*) (*n* = 38, 55.1%), Coleoptera (including *Tenebrio molitor* and other beetles) (*n* = 22, 31.9%), housefly (*Musca domestica*) (*n* = 9, 13.0%), silkworm (*Bombyx mori*) (*n* = 7, 10.1%), and Orthoptera (including crickets and grasshoppers) (*n* = 4, 5.8%) (Figure 2C) (Percentages sum to >100% because some studies evaluated more than one insect category). Among the 69 studies, SGR was reported in 59 studies, WGR in 50 studies, and FCR in 50 studies. Detailed information on each included study is provided in Table S1.

### 3.2. Overall Effect Sizes of Different Insect Meals

**Figure 3:**
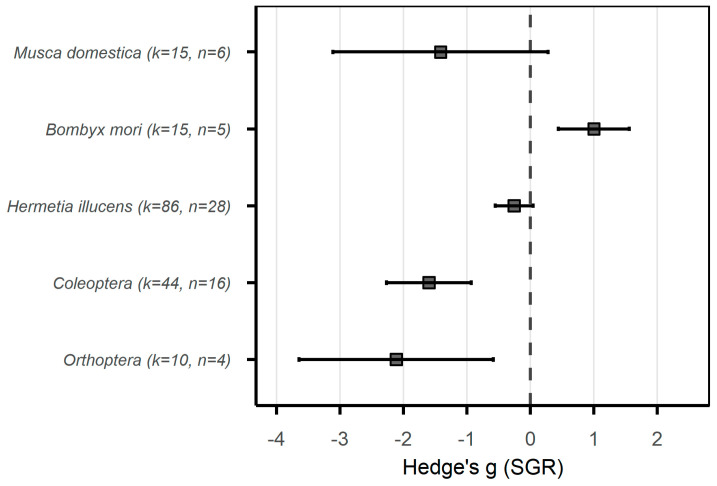
Forest plot of Hedges’ *g* effect sizes (mean and 95% confidence interval) for specific growth rate (SGR) of non-fish aquatic animals fed different insect meals. Squares represent the pooled effect sizes for each subgroup. *n* indicates the number of studies; *k* is the number of comparisons (treatment vs. control group). Insect species: *H. illucens* (black soldier fly), Coleoptera (primarily *T. molitor*, yellow mealworm), *M. domestica* (housefly), *B. mori* (silkworm), and Orthoptera (crickets/grasshoppers).

Different insect meals exerted varying effects on the SGR of non-fish aquatic animals (Figure 3). Among the five insect categories, only *B. mori* meal significantly improved SGR (*p* < 0.05). In contrast, Orthoptera and Coleoptera meals significantly reduced SGR (*p* < 0.05), whereas *H. illucens* and *M. domestica* meals showed no significant effect (*p* > 0.05). High heterogeneity was observed across most subgroups (*I*^2^ > 80%), except for *M. domestica* (*I*^2^ = 0%), indicating the presence of moderating factors.

**Figure 4:**
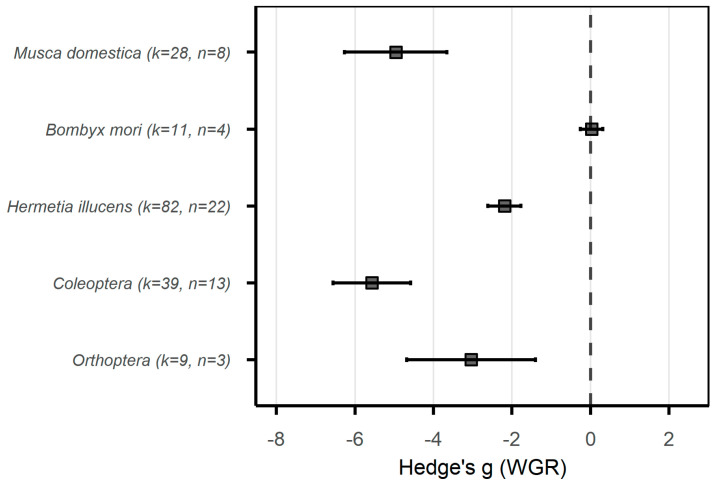
Forest plot of Hedges’ *g* effect sizes (mean and 95% confidence interval) for weight gain rate (WGR) of non-fish aquatic animals fed different insect meals. Squares represent the pooled effect sizes for each subgroup. *n* indicates the number of studies; *k* is the number of comparisons (treatment vs. control group). Insect species as defined in Figure 3.

Regarding WGR (Figure 4), all insect meals except *B. mori* significantly reduced WGR compared with the control diet (*p* < 0.05), with Coleoptera (*g* = −5.57) and *M. domestica* (*g* = −4.96) exhibiting the most pronounced negative effects. *B. mori* meal had no significant impact on WGR (*p* > 0.05). High heterogeneity was detected across all subgroups (*I*^2^ > 87%).

**Figure 5:**
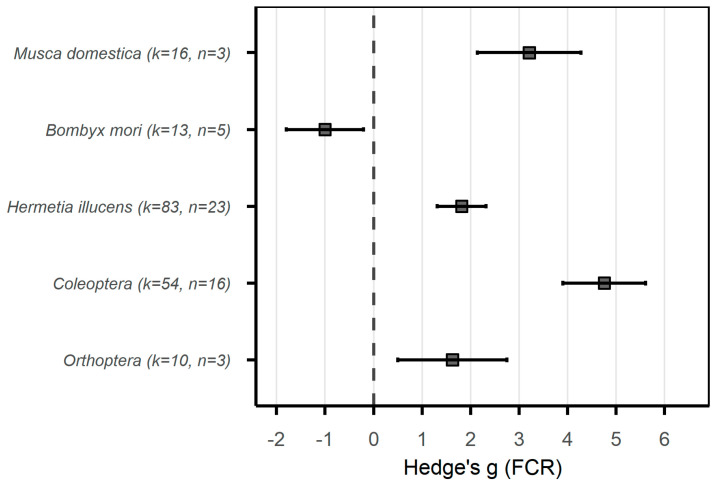
Forest plot of Hedges’ *g* effect sizes (mean and 95% confidence interval) for feed conversion ratio (FCR) of non-fish aquatic animals fed different insect meals. Squares represent the pooled effect sizes for each subgroup. *n* indicates the number of studies; *k* is the number of comparisons (treatment vs. control group). Insect species as defined in Figure 3.

The meta-analysis of FCR revealed that four insect meals significantly increased FCR compared with the control diet (Figure 5), indicating poorer feed utilisation, with Coleoptera (*g* = 4.76, *p* < 0.05) and *M. domestica* (*g* = 3.21, *p* < 0.05) exhibiting the most pronounced negative effects. *H. illucens* and Orthoptera meals also significantly increased FCR (*p* < 0.05), albeit to a lesser extent. In contrast, *B. mori* meal significantly reduced FCR (*g* = −1.00, *p* < 0.05), indicating improved feed utilisation. Substantial heterogeneity was detected across most subgroups (*I*^2^ = 69–91%), suggesting the presence of moderating factors. Detailed effect size estimates for SGR, WGR, and FCR are summarised in Table S2.

### 3.3. Comparison of Effect Sizes Among Insect Meal Categories

To compare the efficacy of different insect meals, Kruskal–Wallis tests were performed on the effect sizes of SGR, WGR, and FCR across the five insect categories (Figures S1–S3). No significant differences were detected among insect meal categories for any growth parameter (SGR: *χ*^2^ = 4.04, *df* = 4, *p* = 0.401; WGR: *χ*^2^ = 4.42, *df* = 4, *p* = 0.352; FCR: *χ*^2^ = 7.68, *df* = 4, *p* = 0.104). Dunn’s post-hoc tests with Bonferroni correction confirmed that none of the pairwise comparisons reached statistical significance (*p* > 0.05 for all).

Numerically, *B. mori* meal tended to exhibit higher SGR and WGR values, whereas *M. domestica* meal showed the highest FCR values among all categories. However, these numerical trends were not statistically supported.

### 3.4. Species-Specific Effects of Major Insect Meals

#### 3.4.1. Black Soldier Fly (*Hermetia illucens*) Meal

**Figure 6:**
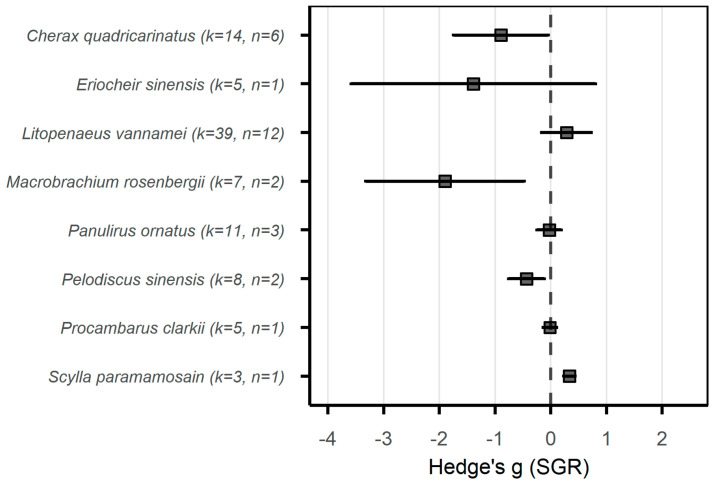
Forest plot of Hedges’ *g* effect sizes (mean and 95% confidence interval) for specific growth rate (SGR) of non-fish aquatic animals fed black soldier fly (*Hermetia illucens*) meal, stratified by host species. Squares represent the pooled effect sizes for each subgroup. *n* indicates the number of studies; *k* is the number of comparisons (treatment vs. control group).

**Figure 7:**
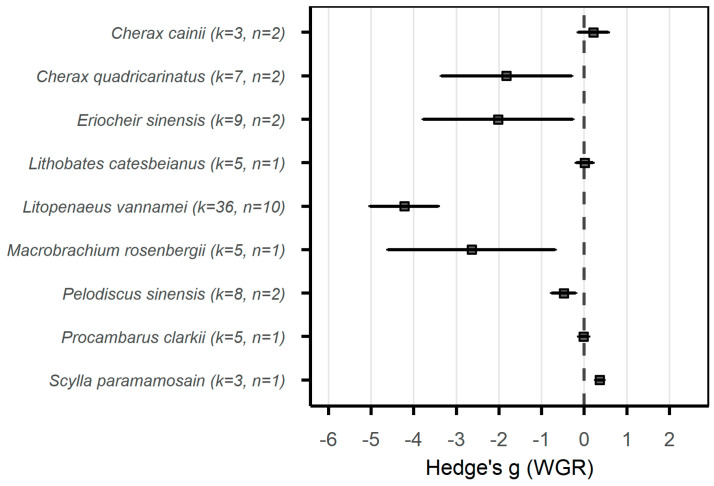
Forest plot of Hedges’ *g* effect sizes (mean and 95% confidence interval) for weight gain rate (WGR) of non-fish aquatic animals fed black soldier fly (*Hermetia illucens*) meal, stratified by host species. Squares represent the pooled effect sizes for each subgroup. *n* indicates the number of studies; *k* is the number of comparisons (treatment vs. control group).

**Figure 8:**
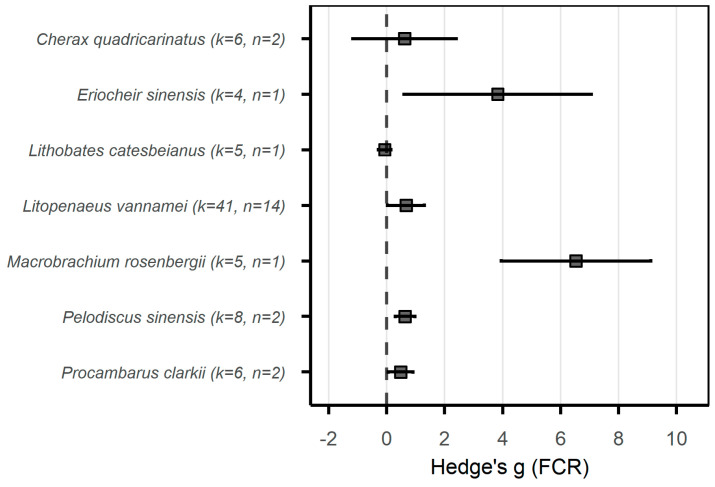
Forest plot of Hedges’ *g* effect sizes (mean and 95% confidence interval) for feed conversion ratio (FCR) of non-fish aquatic animals fed black soldier fly (*Hermetia illucens*) meal, stratified by host species. Squares represent the pooled effect sizes for each subgroup. *n* indicates the number of studies; *k* is the number of comparisons (treatment vs. control group).

*H. illucens* meal exhibited marked species-specific effects (Figure 6, Figure 7 and Figure 8, Table S3). SGR was significantly improved only in mud crab (*Scylla paramamosain*) (*g* = 0.33, *p* < 0.001), but was reduced in several other species, including giant freshwater prawn (*Macrobrachium rosenbergii*) and Chinese soft-shelled turtle (*Pelodiscus sinensis*). WGR followed a similar pattern, being improved only in *S. paramamosain* (*g* = 0.36, *p* < 0.001), but was reduced in several other species, most severely in *L. vannamei* (*g* = −4.23, *p* < 0.001) and *M. rosenbergii* (*g* = −2.65, *p* < 0.01). FCR was increased in five species, with the largest effect observed in *M. rosenbergii* (*g* = 6.53, *p* < 0.001). No significant effects were observed in red swamp crayfish (*Procambarus clarkii*) or American bullfrog (*Lithobates catesbeianus*) for SGR and WGR.

#### 3.4.2. Coleoptera (Primarily *Tenebrio molitor*) Meal

**Figure 9:**
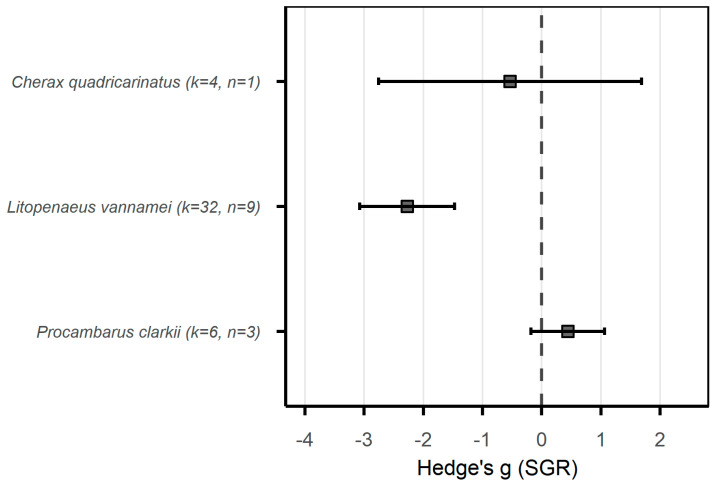
Forest plot of Hedges’ *g* effect sizes (mean and 95% confidence interval) for specific growth rate (SGR) of non-fish aquatic animals fed Coleoptera (primarily *Tenebrio molitor*) meal, stratified by host species. Squares represent the pooled effect sizes for each subgroup. *n* indicates the number of studies; *k* is the number of comparisons (treatment vs. control group).

**Figure 10:**
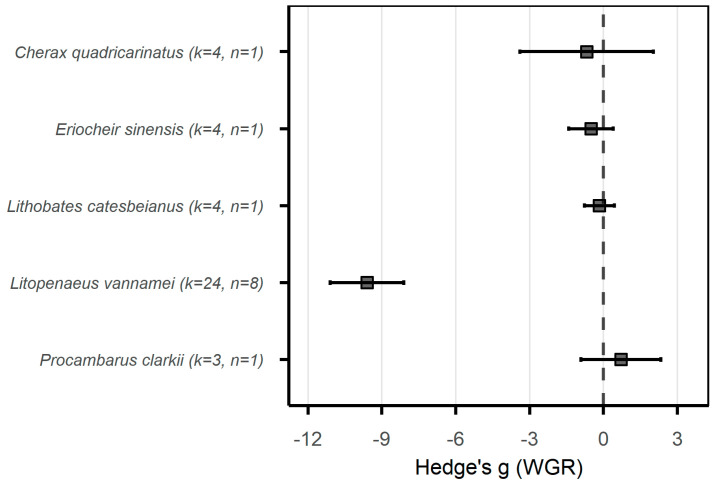
Forest plot of Hedges’ *g* effect sizes (mean and 95% confidence interval) for weight gain rate (WGR) of non-fish aquatic animals fed Coleoptera (primarily *Tenebrio molitor*) meal, stratified by host species. Squares represent the pooled effect sizes for each subgroup. *n* indicates the number of studies; *k* is the number of comparisons (treatment vs. control group).

**Figure 11:**
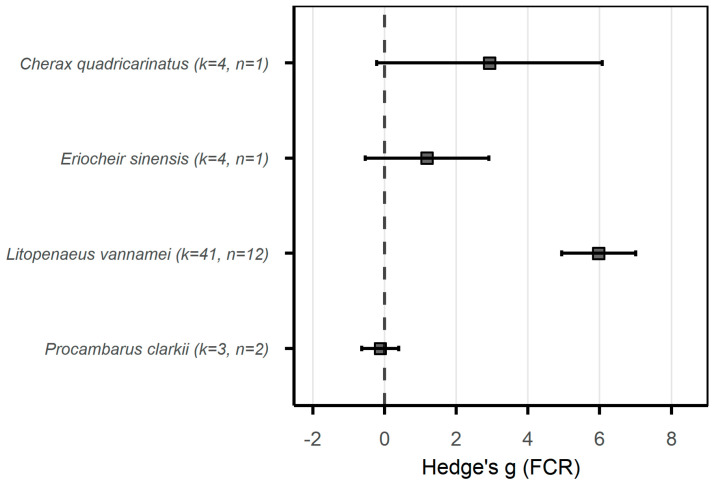
Forest plot of Hedges’ *g* effect sizes (mean and 95% confidence interval) for feed conversion ratio (FCR) of non-fish aquatic animals fed Coleoptera (primarily *Tenebrio molitor*) meal, stratified by host species. Squares represent the pooled effect sizes for each subgroup. *n* indicates the number of studies; *k* is the number of comparisons (treatment vs. control group).

Coleoptera meals also showed significant species-specific effects (Figure 9, Figure 10 and Figure 11, Table S4). SGR and FCR were significantly affected only in *L. vannamei* (SGR: *g* = −2.28, *p* < 0.001; FCR: *g* = 5.97, *p* < 0.001). No significant improvement in WGR was observed across all examined species. WGR was severely reduced in *L. vannamei* (*g* = −9.60, *p* < 0.001). No significant effects were detected in other species (e.g., *P. clarkii*, *Cherax* spp., *L. catesbeianus*).

### 3.5. Meta-Regression of Replacement Level for Major Insect Meals

**Figure 12:**
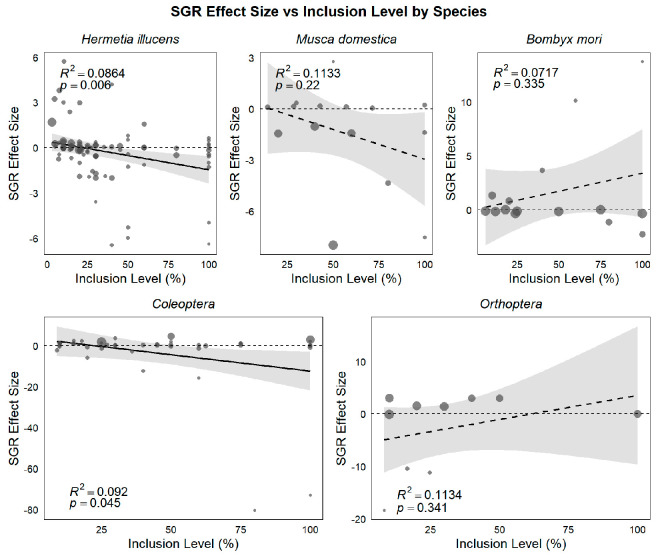
Dose–response relationships between dietary insect meal inclusion level (%) and effect size (Hedges’ *g*) for specific growth rate (SGR). Solid lines indicate significant linear regressions (*p* < 0.05); dashed lines indicate non-significant regressions (*p* > 0.05). Shaded areas represent the 95% confidence intervals of the regression lines. Insect species as defined in Figure 3.

**Figure 13:**
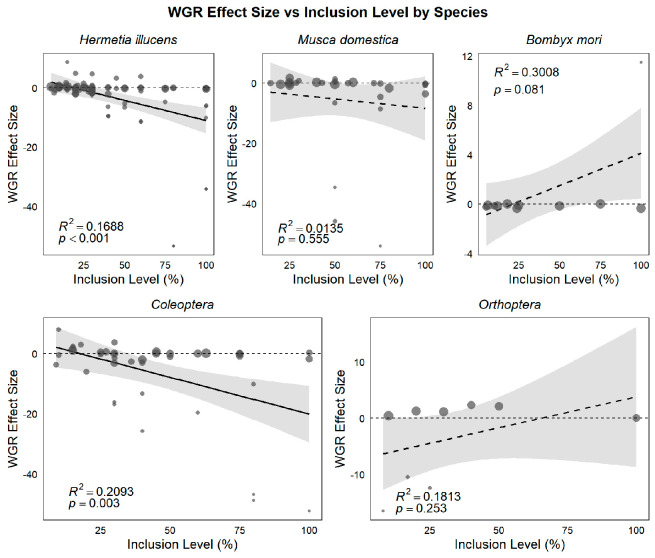
Dose–response relationships between dietary insect meal inclusion level (%) and effect size (Hedges’ *g*) for weight gain rate (WGR). Solid lines indicate significant linear regressions (*p* < 0.05); dashed lines indicate non-significant regressions (*p* > 0.05). Shaded areas represent the 95% confidence intervals of the regression lines. Insect species as defined in Figure 3.

**Figure 14:**
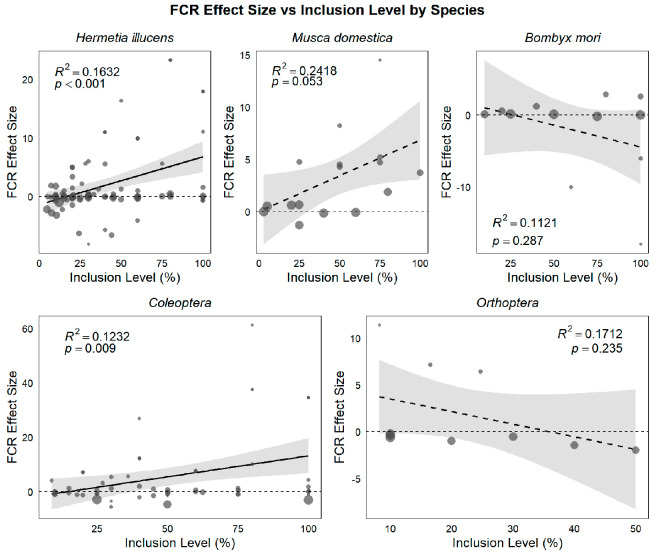
Dose–response relationships between dietary insect meal inclusion level (%) and effect size (Hedges’ *g*) for feed conversion ratio (FCR). Solid lines indicate significant linear regressions (*p* < 0.05); dashed lines indicate non-significant regressions (*p* > 0.05). Shaded areas represent the 95% confidence intervals of the regression lines. Insect species as defined in Figure 3.

Linear meta-regression revealed significant negative correlations between dietary replacement level and growth performance for most insect categories (Figure 12, Figure 13 and Figure 14, Table S5). For *H. illucens* meal, replacement level was significantly negatively correlated with SGR (slope = −0.019, *p* = 0.006) and WGR (slope = −0.137, *p* < 0.001) and positively correlated with FCR (slope = 0.082, *p* < 0.001), indicating progressive impairment of growth and feed utilisation with increasing inclusion. Coleoptera meals showed similar negative correlations with SGR (slope = −0.161, *p* = 0.045) and WGR (slope = −0.244, *p* = 0.003) and a significant positive correlation with FCR (slope = 0.154, *p* = 0.009). For *M. domestica* meal, no significant dose–response relationships were observed for any indicator (*p* > 0.05). No significant dose–response relationships were detected for *B. mori* or Orthoptera across any indicator (*p* > 0.05). Quadratic terms were tested but were not significant for any insect category (*p* > 0.05 for all quadratic coefficients); therefore, linear models were retained.

### 3.6. Meta-Regression of Replacement Level for Litopenaeus vannamei

**Figure 15:**
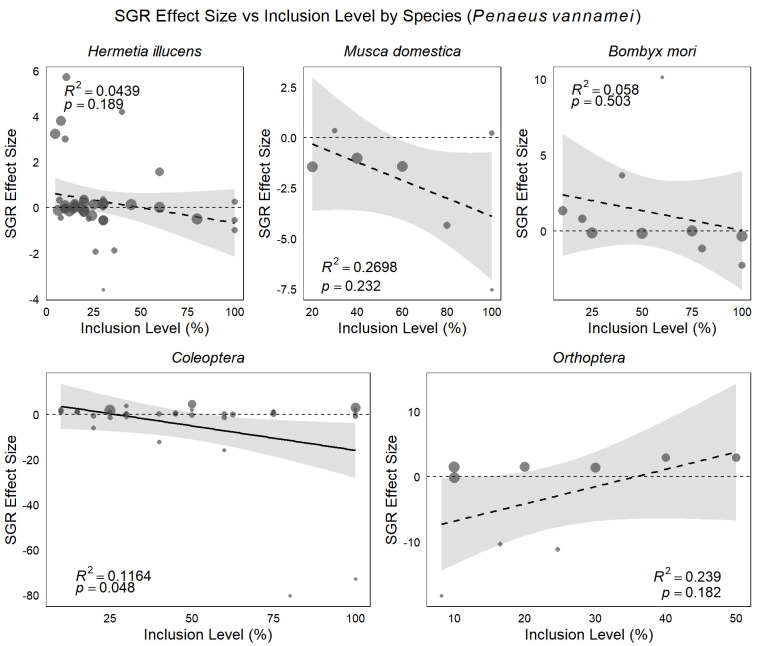
Dose–response relationships between dietary insect meal inclusion level (%) and effect size (Hedges’ *g*) for specific growth rate (SGR) of *L. vannamei.* Solid lines indicate significant linear regressions (*p* < 0.05); dashed lines indicate non-significant regressions (*p* > 0.05). Shaded areas represent the 95% confidence intervals of the regression lines. Insect species as defined in Figure 3.

**Figure 16:**
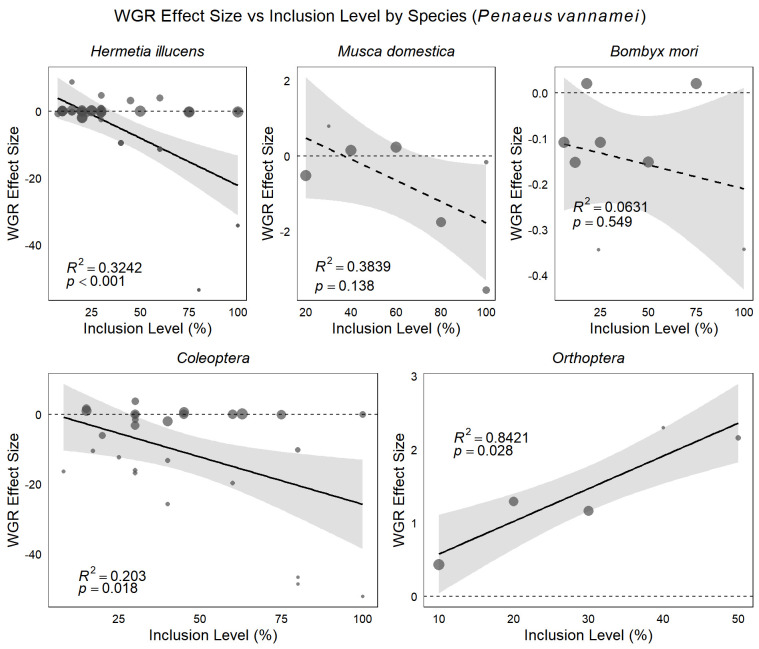
Dose–response relationships between dietary insect meal inclusion level (%) and effect size (Hedges’ *g*) for weight gain rate (WGR) of *L. vannamei.* Solid lines indicate significant linear regressions (*p* < 0.05); dashed lines indicate non-significant regressions (*p* > 0.05). Shaded areas represent the 95% confidence intervals of the regression lines. Insect species as defined in Figure 3.

**Figure 17:**
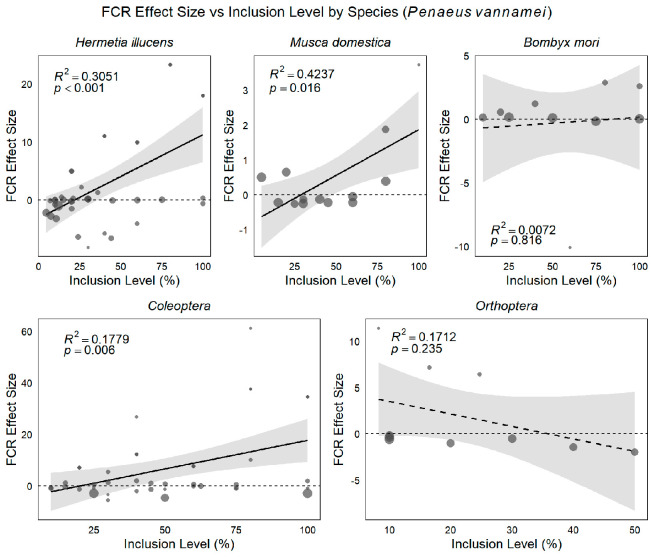
Dose–response relationships between dietary insect meal inclusion level (%) and effect size (Hedges’ *g*) for feed conversion ratio (FCR) of *L. vannamei.* Solid lines indicate significant linear regressions (*p* < 0.05); dashed lines indicate non-significant regressions (*p* > 0.05). Shaded areas represent the 95% confidence intervals of the regression lines. Insect species as defined in Figure 3.

Linear meta-regression for Pacific white shrimp showed that *H. illucens* meal had no significant effect on SGR (*p* = 0.189), but significantly reduced WGR (slope = −0.283, *p* < 0.001) and increased FCR (slope = 0.144, *p* < 0.001). Coleoptera meal significantly reduced SGR (slope = −0.217, *p* = 0.048) and WGR (slope = −0.272, *p* = 0.018) and increased FCR (slope = 0.222, *p* = 0.006). *M. domestica* meal significantly increased FCR (slope = 0.026, *p* = 0.016), but had no significant effects on SGR or WGR (*p* > 0.05). Orthoptera meal showed a significant positive correlation with WGR (slope = 0.045, *p* = 0.028), although based on only five studies. No significant dose–response relationships were detected for *B. mori* meal across any indicator (*p* > 0.05). Quadratic terms were not significant for most combinations; the only significant quadratic relationship (*M. domestica* FCR) was not reported due to the small sample size (*n* = 13) and for consistency (Figure 15, Figure 16 and Figure 17, Table S6).

### 3.7. Publication Bias and Sensitivity Analysis

Egger’s test revealed significant publication bias in most insect meal categories across the three growth indicators (*p* < 0.05), with the exception of *M. domestica* for SGR, Orthoptera for WGR, and *B. mori* for FCR. Trim-and-fill analysis indicated that the adjusted effect sizes remained largely consistent with the original estimates. Sensitivity analysis confirmed that the observed bias was primarily driven by extreme values in a limited number of studies and did not substantially alter the pooled effect estimates for most categories. However, results for Orthoptera should be interpreted cautiously due to the limited number of available studies.

## 4. Discussion

This meta-analysis quantitatively synthesised the effects of insect meals on growth performance in non-fish aquatic animals. *B. mori* meal consistently outperformed other insect meals across all indicators, with significant improvement in SGR and FCR and no adverse effects on WGR, whereas *H. illucens* and Coleoptera meals predominantly impaired performance. Notably, the effects of these two insect meals were consistent across shrimp species but varied among crab species. The outcomes of insect meal substitution in non-fish aquatic animals are therefore jointly determined by insect meal type and host species, underscoring the need for species-specific evaluation.

The consistent positive effects of *B. mori* meal can be attributed to several nutritional and physiological attributes. Its amino acid profile is similar to that of fishmeal and particularly rich in lysine and methionine, which are often limiting in other insect meals [31,32]. *B. mori* also contains ecdysteroids, and their dietary inclusion shortens moulting intervals in *L. vannamei*. Notably, the same study found that even total fishmeal replacement with *B. mori* meal caused no growth impairment [33]. Moreover, the chitin content of *B. mori* is moderate [34], which may confer prebiotic benefits at appropriate inclusion levels [35]. Consistent with these attributes, our meta-regression detected no negative dose–response relationship, indicating that higher substitution levels may be tolerated without compromising growth performance.

*H. illucens* and Coleoptera meals share common nutritional shortcomings that may make them less effective than *B. mori* meal as fishmeal replacements. Both insect meals are naturally deficient in long-chain polyunsaturated fatty acids (LC-PUFAs) such as EPA and DHA, which are abundant in fishmeal [36]. These fatty acids are essential for maintaining cell membrane structure and normal metabolic function in crustaceans [37]. Furthermore, *H. illucens* is rich in saturated fatty acids (predominantly lauric acid), whereas mealworm (*Tenebrio molitor*) contains high total lipid levels [38]. At high dietary inclusion levels, these fatty acid imbalances have been shown to disrupt lipid metabolism in shrimp and crabs [39,40,41].

In addition to these fatty acid-related limitations, both insect meals contain appreciable amounts of chitin [15], a structural polysaccharide that reduces protein digestibility in a dose-dependent manner [42] and, when present at excessive levels, can also impair lipid digestibility in aquatic animals [43]. Additionally, their amino acid profiles are unbalanced compared to fishmeal, with deficiencies in methionine, lysine, and other essential amino acids [44]. Crustaceans have high dietary requirements for these essential amino acids [45], and their deficiency is known to restrain protein synthesis and impair growth [46]. Consistent with these dose-dependent limitations, our meta-regression revealed a negative linear relationship between substitution level and growth performance for both *H. illucens* and Coleoptera meals (Figure 12, Figure 13 and Figure 14).

The strongly species-dependent responses to *H. illucens* and Coleoptera meals (Figure 6, Figure 7, Figure 8, Figure 9, Figure 10 and Figure 11) stem primarily from host-specific physiological differences. The consistent growth depression observed across all shrimp species can be attributed to several physiological characteristics of shrimp, each amplified by the inherent nutritional shortcomings of both insect meals. Shrimp do not increase feed intake in response to declining dietary quality [47]. Consequently, when fishmeal is replaced by insect meals, the effects of lower amino acid availability in insect meals may be amplified in shrimp. This is reflected in our subgroup analysis: Coleoptera meal significantly increased FCR in *L. vannamei* (*g* = 5.97, *p* < 0.001) yet simultaneously depressed growth, a pattern consistent with the absence of compensatory feeding. Moreover, shrimp exhibit limited capacity to digest insect-derived chitin, with an apparent digestibility coefficient of only 28–36% in *L. vannamei*, and excessive dietary chitin (>10%) reduces growth and nutrient digestibility [48]. Apart from these physiological constraints, the fatty acid imbalances common to both insect meals also impose additional metabolic burdens at elevated substitution levels [39,40]. These cumulative constraints align with the dose-dependent growth depression observed in our meta-regression (Figure 12, Figure 13 and Figure 14).

In contrast to the consistent growth depression observed in shrimp, crab species exhibited species-specific responses to both insect meals (Figure 6, Figure 7, Figure 8, Figure 9, Figure 10 and Figure 11). Given the limited sample sizes and the variability across species, any mechanistic interpretation remains tentative. This variability may stem from species-specific differences in dietary ecology. For example, mud crabs (*Scylla* spp.) are active predators [49], whereas Chinese mitten crabs (*Eriocheir sinensis*) are omnivorous with herbivorous–detritivorous tendencies [50]; the digestive physiology shaped by such dietary divergence [51] may influence tolerance to the two insect meals. Future studies with larger sample sizes are needed to clarify these divergent responses. This sharp contrast between shrimp and crabs reinforces the central role of host physiology in determining the outcomes of insect meal substitution.

Pacific white shrimp (*Litopenaeus vannamei*) was the most data-rich species in our dataset. The species-specific meta-regression for *L. vannamei* (Figure 15, Figure 16 and Figure 17) revealed significant negative linear dose–response relationships across multiple indicators for Coleoptera meals (all three growth indicators) and *H. illucens* meals (WGR and FCR). These results indicate that growth suppression and feed efficiency impairment worsen progressively with increasing inclusion levels. Based on these trends, and given that independent feeding trials have reported growth depression when inclusion exceeds the 25–30% range [52,53], we recommend a conservative upper limit of 25–30% for these two insect meals in *L. vannamei* feeds. In contrast, *B. mori* meal exhibited no significant dose–response relationship, reinforcing its favourable nutritional profile and higher tolerance to elevated inclusion levels noted above. For *M. domestica* and Orthoptera meals, the limited number of studies precludes firm recommendations, although the observed trends suggest caution at higher substitution rates. These dose–response patterns underscore the importance of insect-specific and host-specific considerations in aquafeed formulation.

These findings complement the fish-centric meta-analyses by Hua [20] and Tran et al. [22] and together provide a broader view of insect meal effects across aquatic taxa. The divergent response to *Tenebrio molitor* meal, broadly effective in fish yet detrimental in shrimp, underscores species-specific sensitivities that warrant further investigation. In contrast, the consistent benefits of *B. mori* meal across both fish and non-fish taxa highlight its broad nutritional value.

The substantial heterogeneity observed across studies can be partly attributed to the moderators examined, including insect meal type, host species, and substitution level. Some of the remaining variation likely stems from factors that could not be systematically assessed, such as experimental duration and basal diet composition. Publication bias was detected in some subgroup analyses, but sensitivity analyses indicated that effect estimates remained largely unchanged. For black soldier fly and Coleoptera meals, species-level subgroup analyses were feasible because these were the only categories with sufficient cross-species data (three or more host species, each with three or more effect sizes). In contrast, for housefly, silkworm, and Orthoptera meals, the available effect sizes were predominantly derived from a single host species (*L. vannamei*, accounting for over 70% of effect sizes in most cases). Formal species-level stratification was therefore statistically impractical for these three categories, as the resulting subgroups would lack sufficient power for reliable inference. Consequently, the limited data for certain non-fish species constrained robust species-specific inferences, and the substitution gradients employed in the included studies varied widely. Given the unique physiological constraints of *L. vannamei*, the dose–response thresholds obtained from this species may not generalise to other crustaceans, despite the richness of the dataset. Future studies should therefore employ standardised substitution gradients across a broader range of non-fish species and include more data on underrepresented taxa such as crabs, turtles, and frogs to establish species-specific safe inclusion limits and further investigate the physiological basis of host-dependent responses to insect meal substitution.

Based on this quantitative synthesis, *B. mori* meal emerges as the most effective insect protein for growth performance among those evaluated in non-fish aquatic animals. However, as a by-product of the silk industry, its supply is geographically concentrated in a few Asian countries [54], and product standardisation remains an unresolved challenge for large-scale application [55]. In contrast, *H. illucens* and yellow mealworm (*Tenebrio molitor*) meals benefit from established large-scale rearing systems and are the most widely studied insect species for aquafeed use [19,56]. Partial defatting improves the suitability of both meals for shrimp feeds, as demonstrated for *H. illucens* larvae meal [57] and *Tenebrio molitor* meal [58]. Given the consistent growth suppression observed in shrimp at high substitution levels of these two insect meals, a conservative substitution limit of 25–30% is advisable. Where economically viable, mixed-protein strategies, combining insect meal with complementary protein sources such as poultry by-product meal or rapeseed meal, have been shown to sustain shrimp growth without fishmeal [59], offering a practical approach to reduce feed costs while mitigating the nutritional shortcomings of single-insect-meal substitution. For crabs, the feasibility of insect meal inclusion appears more species-dependent, and greater caution is warranted. The economic viability of these strategies under commercial aquaculture conditions requires further investigation.

## 5. Conclusions

This systematic review and meta-analysis quantitatively synthesised the effects of insect meals as fishmeal replacements in non-fish aquatic animals based on 69 included studies [33,48,52,57,58,60,61,62,63,64,65,66,67,68,69,70,71,72,73,74,75,76,77,78,79,80,81,82,83,84,85,86,87,88,89,90,91,92,93,94,95,96,97,98,99,100,101,102,103,104,105,106,107,108,109,110,111,112,113,114,115,116,117,118,119,120,121,122,123]. Among the five insect categories evaluated, *B. mori* meal consistently demonstrated favourable growth performance, with no significant impairment of growth or feed utilisation across all indicators. In contrast, *H. illucens* and Coleoptera (primarily *T. molitor*) meals exhibited dose-dependent negative effects on growth performance, particularly in shrimp species. Subgroup analyses revealed pronounced host-specific responses, with shrimp showing consistent growth depression, whereas certain crab species displayed tolerance or even improved performance. These findings underscore that the efficacy of insect meal substitution is jointly governed by insect type and host physiology, highlighting the necessity of species-specific evaluation in aquafeed formulation. *B. mori* meal represents a promising alternative to fishmeal for non-fish aquaculture, while *H. illucens* and Coleoptera meals require careful inclusion level management to avoid compromising production performance.

## Data Availability

The original contributions presented in this study are included in the article/Supplementary Material. Further inquiries can be directed to the corresponding author.

## Supplementary Materials

The following supporting information can be downloaded at https://www.mdpi.com/article/10.3390/insects17070699/s1: Figure S1: Comparison of SGR effect sizes among different insect meal categories; Figure S2: Comparison of WGR effect sizes among different insect meal categories; Figure S3: Comparison of FCR effect sizes among different insect meal categories; Table S1: References included in the quantitative meta-analysis; Table S2: Hedges’ g effect size summary for SGR, WGR and FCR by insect meal category; Table S3: Hedges’ g effect size summary for SGR, WGR and FCR of aquatic species fed dietary *Hermetia illucens* meal; Table S4: Hedges’ g effect size summary for SGR, WGR and FCR of aquatic species fed dietary Coleoptera meal; Table S5: Summary of linear meta-regression between replacement level and effect size for SGR, WGR and FCR across all species; Table S6: Summary of linear meta-regression between replacement level and effect size for SGR, WGR and FCR in *Litopenaeus vannamei*; Supplementary S1: References of the 69 studies included in the meta-analysis; PRISMA 2020 checklist.
