# Supplementary material for: Effects of Fishmeal Replacement with Insect Meals on Growth Performance in Non-Fish Aquatic Animals: A Meta-Analysis
Source: Insects. 2026 Jul 6;17(7):699. doi: 10.3390/insects17070699 (PMC13409791; doi:10.3390/insects17070699)
Supplement: Supplementary file 1 [file insects-17-00699-s001.zip › PRISMA_2020_checklist.pdf]

PRISMA 2020 Main Checklist

Topic	No.	Item	Location where item is reported
**TITLE**			
**Title**	1	Identify the report as a systematic review.	Title
**ABSTRACT**			
**Abstract**	2	See the PRISMA 2020 for Abstracts checklist	Abstract
**INTRODUCTION**			
**Rationale**	3	Describe the rationale for the review in the context of existing knowledge.	1. Introduction, paragraphs 1-3
**Objectives**	4	Provide an explicit statement of the objective(s) or question(s) the review addresses.	1. Introduction, last paragraph
**METHODS**			
**Eligibility criteria**	5	Specify the inclusion and exclusion criteria for the review and how studies were grouped for the syntheses.	2.1 Literature search
**Information sources**	6	Specify all databases, registers, websites, organisations, reference lists and other sources searched or consulted to identify studies. Specify the date when each source was last searched or consulted.	2.1 Literature search
**Search strategy**	7	Present the full search strategies for all databases, registers and websites, including any filters and limits used.	2.1 Literature search
**Selection process**	8	Specify the methods used to decide whether a study met the inclusion criteria of the review, including how many reviewers screened each record and each report retrieved, whether they worked independently, and if applicable, details of automation tools used in the process.	2.1 Literature search and 2.2 Data extraction and assessment
**Data collection process**	9	Specify the methods used to collect data from reports, including how many reviewers collected data from each report, whether they worked independently, any processes for obtaining or confirming data from study investigators, and if applicable, details of automation tools used in the process.	2.2 Data extraction and assessment
**Data items**	10a	List and define all outcomes for which data were sought. Specify whether all results that were compatible with each outcome domain in each study were sought (e.g. for all measures, time points, analyses), and if not, the methods used to decide which results to collect.	2.3 Data analysis

Topic	No.	Item	Location where item is reported
	10b	List and define all other variables for which data were sought (e.g. participant and intervention characteristics, funding sources). Describe any assumptions made about any missing or unclear information.	2.2 Data extraction and assessment
**Study risk of bias assessment**	11	Specify the methods used to assess risk of bias in the included studies, including details of the tool(s) used, how many reviewers assessed each study and whether they worked independently, and if applicable, details of automation tools used in the process.	Not assessed (only publication bias was evaluated; see section 2.4 Heterogeneity and publication bias)
**Effect measures**	12	Specify for each outcome the effect measure(s) (e.g. risk ratio, mean difference) used in the synthesis or presentation of results.	2.3 Data analysis（“Hedges' g effect size was calculated”）
**Synthesis methods**	13a	Describe the processes used to decide which studies were eligible for each synthesis (e.g. tabulating the study intervention characteristics and comparing against the planned groups for each synthesis (item 5)).	2.1 (Literature search) and 2.3 Data analysis (subgroup analyses by insect meal category)
	13b	Describe any methods required to prepare the data for presentation or synthesis, such as handling of missing summary statistics, or data conversions.	2.2 Data extraction and assessment
	13c	Describe any methods used to tabulate or visually display results of individual studies and syntheses.	Results section (Figures 3–17, S1–S3)
	13d	Describe any methods used to synthesize results and provide a rationale for the choice(s). If meta-analysis was performed, describe the model(s), method(s) to identify the presence and extent of statistical heterogeneity, and software package(s) used.	2.3 Data analysis and 2.4 Heterogeneity and publication bias
	13e	Describe any methods used to explore possible causes of heterogeneity among study results (e.g. subgroup analysis, meta-regression).	2.3 Data analysis (subgroup analyses and meta-regression)
	13f	Describe any sensitivity analyses conducted to assess robustness of the synthesized results.	2.4 Heterogeneity and publication bias (sensitivity analysis)
**Reporting bias assessment**	14	Describe any methods used to assess risk of bias due to missing results in a synthesis (arising from reporting biases).	2.4 Heterogeneity and publication bias (funnel plots, Egger's test, trim-and-fill)
**Certainty assessment**	15	Describe any methods used to assess certainty (or confidence) in the body of evidence for an outcome.	Not assessed
**RESULTS**			
**Study selection**	16a	Describe the results of the search and selection process, from the number of records identified in the search to the number of studies included in the review, ideally using a flow diagram.	Figure 1 and Section 3.1 Overview of included studies

Topic	No.	Item	Location where item is reported
	16b	Cite studies that might appear to meet the inclusion criteria, but which were excluded, and explain why they were excluded.	Figure 1 (PRISMA flow diagram)
**Study characteristics**	17	Cite each included study and present its characteristics.	Table S1 (Supplementary Material) and Section 3.1
**Risk of bias in studies**	18	Present assessments of risk of bias for each included study.	Not assessed (only publication bias was evaluated)
**Results of individual studies**	19	For all outcomes, present, for each study: (a) summary statistics for each group (where appropriate) and (b) an effect estimate and its precision (e.g. confidence/credible interval), ideally using structured tables or plots.	Figures 3–11; Tables S2–S4
**Results of syntheses**	20a	For each synthesis, briefly summarise the characteristics and risk of bias among contributing studies.	Characteristics of studies contributing to each synthesis are summarized in Section 3.1 and detailed in Table S1. Individual study risk of bias was not assessed; publication bias for each synthesis is reported in Section 3.7.
	20b	Present results of all statistical syntheses conducted. If meta-analysis was done, present for each the summary estimate and its precision (e.g. confidence/credible interval) and measures of statistical heterogeneity. If comparing groups, describe the direction of the effect.	Sections 3.2–3.6; Tables S2–S6; Figures 3–17
	20c	Present results of all investigations of possible causes of heterogeneity among study results.	Sections 3.4 (species-specific effects) and 3.5–3.6 (meta-regression)
	20d	Present results of all sensitivity analyses conducted to assess the robustness of the synthesized results.	Section 3.7 Publication bias and sensitivity analysis
**Reporting biases**	21	Present assessments of risk of bias due to missing results (arising from reporting biases) for each synthesis assessed.	Section 3.7 Publication bias and sensitivity analysis
**Certainty of evidence**	22	Present assessments of certainty (or confidence) in the body of evidence for each outcome assessed.	Not assessed
**DISCUSSION**			
**Discussion**	23a	Provide a general interpretation of the results in the context of other evidence.	Section 4. Discussion (especially paragraphs comparing with fish meta-analyses)
	23b	Discuss any limitations of the evidence included in the review.	Section 4. Discussion (limitations paragraph)
	23c	Discuss any limitations of the review processes used.	Section 4. Discussion (limitations paragraph)
	23d	Discuss implications of the results for practice, policy, and future research.	Section 4. Discussion (last two paragraphs)

Topic	No.	Item	Location where item is reported
**OTHER INFORMATION**			
**Registration and protocol**	24a	Provide registration information for the review, including register name and registration number, or state that the review was not registered.	Not registered
	24b	Indicate where the review protocol can be accessed, or state that a protocol was not prepared.	No protocol was prepared
	24c	Describe and explain any amendments to information provided at registration or in the protocol.	Not applicable
**Support**	25	Describe sources of financial or non-financial support for the review, and the role of the funders or sponsors in the review.	Funding section
**Competing interests**	26	Declare any competing interests of review authors.	Conflicts of Interest section
**Availability of data, code and other materials**	27	Report which of the following are publicly available and where they can be found: template data collection forms; data extracted from included studies; data used for all analyses; analytic code; any other materials used in the review.	Data Availability Statement; data are available in the article and supplementary materials.

From: Page MJ, McKenzie JE, Bossuyt PM, Boutron I, Hoffmann TC, Mulrow CD, et al. The PRISMA 2020 statement: an updated guideline for reporting systematic reviews. MetaArXiv. 2020, September 14. DOI: 10.31222/osf.io/v7gm2. For more information, visit: www.prisma-statement.org
