# Supplementary material for: Effects of Fishmeal Replacement with Insect Meals on Growth Performance in Non-Fish Aquatic Animals: A Meta-Analysis
Source: Insects. 2026 Jul 6;17(7):699. doi: 10.3390/insects17070699 (PMC13409791; doi:10.3390/insects17070699)
Supplement: Supplementary file 1 [file insects-17-00699-s001.zip › Supplementary_Tables_and_Content.pdf]

Supplementary Material

Figure S1. Comparison of SGR effect sizes among different insect meal categories.

Figure S2. Comparison of WGR effect sizes among different insect meal categories.

Figure S3. Comparison of FCR effect sizes among different insect meal categories.

Table S1. References included in the quantitative meta-analysis.

Table S2. Hedges' *g* effect size summary for SGR, WGR and FCR by insect meal category.

Table S3. Hedges' *g* effect size summary for SGR, WGR and FCR of aquatic species fed dietary *Hermetia illucens* meal.

Table S4. Hedges' *g* effect size summary for SGR, WGR and FCR of aquatic species fed dietary Coleoptera meal.

Table S5. Summary of linear meta-regression between replacement level and effect size for SGR, WGR and FCR across all species.

Table S6. Summary of linear meta-regression between replacement level and effect size for SGR, WGR and FCR in *LitoPenaeus vannamei*.

Supplementary S1. References of the 69 studies included in the meta-analysis.

Table S1. References included in the quantitative meta-analysis.

References	Year of publication	Country	Aquaculture species		Fed insect meals		*Replaced ingredient*	*Replacement levels（%）*	Duration *(days)*
			Common name	*Latin name*	*Common name*	*Scientific name*			
1	2021	Malaysia	whiteleg shrimp	*LitoPenaeus vannamei*	black cricket	*Gryllus bimaculatus*	fish meal	0,10,20,30,40,50	40
2	2024	Australia	Marron crayfish	*Cherax cainii*	Black soldier fly	*Hermetia illucens*	fish meal	0,10	110
3	2021	China	Chinese soft-shelled turtle	*Pelodiscus sinensis*	Black soldier fly	*Hermetia illucens*	defatted black soldier fly larvae meal	0,5,10,15,20	45
4	2019	Australia	Marron	*Cherax cainii*	Black soldier fly	*Hermetia illucens*	fish meal	0,27	60
5	2021	South Korea	Pacific white shrimp	*LitoPenaeus vannamei*	Mealworm	*Tenebrio molitor*	Fishmeal (tuna byproduct)	0,10	65
					Silkworm	*Bombyx mori*		0,10	
					Black soldier fly	*Hermetia illucens*		0,10	
					Rice grasshopper	*Oxya chinensis*		0,10	
					Two-spotted cricket	*Gryllus bimaculatus*		0,10	
					Dynastid beetle	*Allomyrina dichotoma*		0,10	
					White-spotted flower chafer	*Protaetia brevitarsis*		0,10	
6	2022	China	Pacific White Shrimp	*LitoPenaeus vannamei*	black soldier fly	*Hermetia illucens*	fishmeal	30	30
					Tenebrio molitor	*Tenebrio molitor*		30	
7	2024	China	Red Swamp Crayfish	*Procambarus clarkii*	*Tenebrio molitor*	*Tenebrio molitor*	fishmeal	10,15	84
8	2019	Thailand	Pacific White Shrimp	*LitoPenaeus vannamei*	*Tenebrio molitor*	*Tenebrio molitor*	fishmeal	0,25,50,75,100	56
9	2023	Switzerland	shrimp	*LitoPenaeus vannamei*	black soldier fly	*Hermetia illucens*	fish meal	0,25,50,75,100	42

10	2023	Malaysia	lobster	*Panulirus ornatus*	black soldier fly	*Hermetia illucens*	fish meal	0,25,35,50	56
11	2023	Iran	Pacific white shrimp	*LitoPenaeus vannamei*	mealworm	*Tenebrio molitor*	fish meal	0,15,30,60,100	60
12	2021	France	Pacific White Shrimp	*LitoPenaeus vannamei*	Black soldier fly	*Hermetia illucens*	fishmeal	0,4.5,7.5,10.5	28
13	2020	South Korea	Pacific White Shrimp	*LitoPenaeus vannamei*	Black soldier fly,	*Hermetia illucens,*	fishmeal	0,20,40,60	57
					mealworm	*Tenebrio molitor*		0,20,40,60	
14	2024	China	Pacific White Shrimp	*LitoPenaeus vannamei*	Black Soldier Fly	*Hermetia illucens*	fish meal	0,20,40,60,80,100	56
15	2023	China	Pacific white shrimp	*LitoPenaeus vannamei*	black soldier fly	*Hermetia illucens*	fish meal	0,20,40,60,80,100	56
					Yellow mealworm	*Tenebrio molitor*		0,20,40,60,80,100	
					Tenebrio molitor	*Tenebrio molitor*		0,40,80	
					Tenebrio molitor	*Tenebrio molitor*		0,40,80	
16	2023	China	Macrobrachium rosenbergii	*Macrobrachium rosenbergii*	black fly larvae	*Hermetia illucens*	fish meal	0,10,20,30,40,50	56
17	2023	China	*Scylla paramamosain*	*Scylla paramamosain*	defatted black soldier fly	*Hermetia illucens*	fish meal	0,25,50,75	28
18	2023	China	juvenile soft-shelled turtle	*Pelodiscus sinensis*	defatted black soldier fly larve	*Hermetia illucens*	fish meal	0,15,30,45,60	56
19	2023	China	Pacific white shrimp	*LitoPenaeus vannamei*	*Tenebrio molitor*	*Tenebrio molitor*	fish meal	0,20,40,60,80,100	56
20	2019	China	Bullfrog	*Lithobates catesbeianus*	*Tenebrio molitor*	*Tenebrio molitor*	fish meal	0,25,50,75,100	56
21	2017	China	juvenile soft-shelled turtles	*Pelodiscus sinensis*	silkworm pupa	*Bombyx mori*	white fish meal	0,5,10	56
22	2024	China	Juvenile Chinese Mitten Crab	*Eriocheir sinensis*	Black Soldier Fly	*Hermetia illucens*	Fish meal	0,10,20,30,40,50	56
23	2023	China	mud crab	*Scylla paramamosain*	zymolytic black soldier fly larvae	*Hermetia illucens*	Fish meal	0,5,10,15	56
24	2021	Mexico	Bullfrog tadpole	*Lithobates catesbeianus*	Housefly larvae	*Musca domestica*	Fish meal	0,25,50,75	112
			Bullfrog	*Lithobates catesbeianus*	Housefly larvae	*Musca domestica*	Fish meal	0,25,50,75	

25	2012	China	juvenile white shrimp	*LitoPenaeus vannamei*	housefly maggot	*Musca domestica*	Fish meal	0,20,40,60,80,100	45
26	2023	China	juvenile white shrimp	*LitoPenaeus vannamei*	black soldier fly	*Hermetia illucens*	Fish meal	0,10,20,30	49
27	2012	China	Red swamp crayfish	*Procambarus clarkii*	Maggots, mealworms, locusts	*Musca domestica, Tenebrio molitor, Orthoptera*	Fish meal	0,100	70
28	2023	China	Red swamp crayfish	*Procambarus clarkii*	black soldier fly	*Hermetia illucens*	Fish meal	0,20,40,60,80,100	90
29	2019	China	juvenile white shrimp	*LitoPenaeus vannamei*	black soldier fly	*Hermetia illucens*	Fish meal	0,10,15,20,25,30	56
30	2017	China	white shrimp	*LitoPenaeus vannamei*	defatted silkworm pupa	*Bombyx mori*	fish meal	0,6,12,18,24	56
31	2022	China	Mud crab	*Scylla paramamosain*	Black soldier fly	*Hermetia illucens*	fish meal	0, 25, 50, 75, 100	60
32	2022	China	Pacific white shrimp	*LitoPenaeus vannamei*	Mealworm	*defatted Tenebrio molitor*	fish meal	0,30	42
33	2023	Italy	freshwater prawn	*Macrobrachium rosenbergii*	black soldier fly	*Hermetia illucens*	fish meal	0,3,20	60
34	2013	China	Soft-shell Turtle	*Pelodiscus sinensis Japanese strain*	housefly maggot	*Musca domestica*	fish meal	0,50	270
35	2007	China	Pacific white shrimp	*LitoPenaeus vannamei*	Housefly maggot	*Musca domestica*	Fish meal	0,3,5	56
36	2017	China	Chinese soft-shelled turtle	*Pelodiscus sinensis*	housefly maggot	*Musca domestica*	fish meal	0,50,100	30
37	2025	Mexico	Pacific white shrimp	*LitoPenaeus vannamei*	cricket	*Acheta domesticus*	fish meal	0,8.2,16.5,24.7	64
38	2025	India	Pacific white shrimp	*LitoPenaeus vannamei*	Black Soldier Fly	*Hermetia illucens*	fish meal	0, 100	60
39	2020	Australia	freshwater crayfish	*Cherax cainii*	black soldier fly	*Hermetia illucens*	fish meal	0,100	56
40	2021	China	Pacific white shrimp	*LitoPenaeus vannamei*	Black Soldier Fly	*Hermetia illucens*	fish meal	0,25,50,75,100	45
41	2019	China	Pacific white shrimp	*LitoPenaeus vannamei*	Silkworm pupae	*Bombyx mori*	fish meal	0,25,50,75,100	56

42	2011	India	Himalayan prawn	*Macrobrachium dayanum*	Silkworm pupae	*Bombyx mori*	fish meal	0,100	90
43	2023	India	Pacific white shrimp	*LitoPenaeus vannamei*	silkworm pupae	*Bombyx mori L.*	fish meal	0,20,40,60,80,100	45
44	2024	China	Chinese mitten crab	*Eriocheir sinensis*	defatted yellow mealworm	*Tenebrio molitor*	fish meal	0,25,50,75,100	56
45	2023	Greece	Freshwater crayfish	*Pontastacus leptodactylus*	Black soldier fly	*Hermetia illucens*	fish meal	0,50,100	98
46	2021	China	Pacific white shrimp	*LitoPenaeus vannamei*	black soldier fly larvae	*Hermetia illucens*	fish meal	0,10,20,30	49
47	2024	China	Redclaw Crayfish	*Cherax quadricarinatus*	Black Soldier Fly Larvae	*Hermetia illucens*	fish meal	0,100	56
48	2023	China	Pacific white shrimp	*LitoPenaeus vannamei*	Yellow mealworm	*Tenebrio molitor*	fish meal	63	56
49	2022	China	Redclaw Crayfish	*Cherax quadricarinatus*	Yellow mealworm	*Tenebrio molitor*	fish meal	0,9,18,27,36	60
					black soldier fly larvae	*Hermetia illucens*	fish meal	0,7,14,21,28	
50	2021	China	Pacific white shrimp	*LitoPenaeus vannamei*	Defatted black soldier fly larvae	*Hermetia illucens*	fish meal	0,15,30,45,60,80	56
51	2021	Turkey	Narrow-clawed crayfish	*Pontastacus leptodactylus*	yellow mealworm	*Tenebrio molitor*	fish meal	0,50,100	80
52	2017	Brazil	Pacific white shrimp	*LitoPenaeus vannamei*	Yellow mealworm	*Tenebrio molitor*	fish meal	0,25,50,75,100	42
53	2023	China	Pacific white shrimp	*LitoPenaeus vannamei*	Yellow mealworm	*Tenebrio molitor*	fish meal	0,15,30,45	66
54	2022	China	Pacific white shrimp	*LitoPenaeus vannamei*	Yellow mealworm	*Tenebrio molitor*	fish meal	0,30	42
55	2022	China	Chinese soft-shelled turtles	*Pelodiscus sinensis*	The black soldier fly larvae	*Hermetia illucens*	fish meal	0,5,10,15,20	70
56	2025	China	Pacific white shrimp	*LitoPenaeus vannamei*	Defatted black soldier fly larvae	*Hermetia illucens*	fish meal	0,15,30,45,60,75	56
57	2017	Brazil	Pacific white shrimp	*LitoPenaeus vannamei*	Yellow mealworm	*Tenebrio molitor*	fish meal	0,25,50,75,100	42
58	2020	Greece	Baltic prawns	*Palaemon adspersus*	Yellow mealworm	*Tenebrio molitor*	fish meal	0,30	60
					The black soldier fly larvae	*Hermetia illucens*		0,30	

					Housefly maggot	*Musca domestica*		0,30	
59	2024	Thailand	Pacific white shrimp	*LitoPenaeus vannamei*	The black soldier fly larvae	*Hermetia illucens*	fish meal	0,12,24,44	45
60	2021	Indonesia	Pacific white shrimp	*LitoPenaeus vannamei*	The black soldier fly larvae	*Hermetia illucens*	fish meal	0,7.5,15,22.5	49
61	2024	Indonesia	Juvenile Lobster	*Panulirus ornatus*	The black soldier fly larvae	*Hermetia illucens*	fish meal	0,25	56
					fish protein hydrolysate	fish protein hydrolysate	fish meal	0,25	
					Full-fat black soldier fly larvae	*Hermetia illucens*	fish meal	0,25	
					Defatted black soldier fly larvae	*Hermetia illucens*	fish meal	0,50	
					Dietary black soldier fly larvae	*Hermetia illucens*	fish meal	0,50	
62	2023	China	Pacific white shrimp	*LitoPenaeus vannamei*	Superworm	*Zophobas atratus*	fish meal	0,15,30,45,60,75,100	56
63	2018	South Korea	Pacific white shrimp	*LitoPenaeus vannamei*	Mealworm	*Tenebrio molitor*	fish meal	0,25,50,100	56
64	2017	USA	Pacific white shrimp	*LitoPenaeus vannamei*	Black soldier fly	*Hermetia illucens*	fish meal	0,7,14,21,26,36	63
65	2004	India	Freshwater Prawn	*Macrobrachium malcolmsonii*	Silkworm pupae	*Bombyx mori*	fish meal	0,100	60
66	2024	China	Chinese mitten crab	*Eriocheir sinensis*	black soldier fly	*Hermetia illucens*	fish meal	0,25,50,75,100	56
67	2018	China	Bullfrog	*Lithobates catesbeianus*	Housefly maggot	*Musca domestica*	fish meal	0,25,50,75,100	56
68	2025	China	Bullfrog	*Lithobates catesbeianus*	Black soldier fly	*Hermetia illucens*	fish meal	0,20,40,60,80,100	56
69	2025	China	Chinese mitten crab	*Eriocheir sinensis*	Housefly maggot	*Musca domestica*	fish meal	0,14,29,43,57,71	56

Table S2. Hedges' *g* effect size summary for specific growth rate (SGR), weight gain rate (WGR), and feed conversion ratio (FCR) by insect meal category.

**Insect meal category**	**indicator**	**n**	**k**	Hedges'g	95% Cl(Lower)	95% Cl(Upper)	**P(effect)**	**I^2(%)**	**Q**	**p(Q)**	**Egger's test p**
Orthoptera	SGR	4	10	-2.11	-3.65	-0.58	<0.05	88.45	77.92	<0.001	0.0109
Black soldier fly		28	86	-0.26	-0.56	0.04	>0.05	45.74	156.66	<0.001	0.0176
Coleoptera		16	44	-1.60	-2.27	-0.94	<0.05	95.87	1040.97	<0.001	<0.0001
Silkworm		5	15	1.00	0.44	1.56	<0.05	89.99	139.85	<0.001	0.0086
Housefly		6	15	-1.42	-3.12	0.28	>0.05	0.00	11.05	0.68	0.0628
Orthoptera	WGR	3	9	-3.04	-4.68	-1.40	<0.05	87.49	63.96	<0.001	0.0616
Black soldier fly		22	81	-2.21	-2.63	-1.78	<0.05	93.93	1317.53	<0.001	<0.0001
Coleoptera		13	39	-5.57	-6.55	-4.59	<0.05	94.14	649.00	<0.001	<0.0001
Silkworm		4	11	0.03	-0.26	0.32	>0.05	89.80	98.08	<0.001	0.0012
Housefly		8	28	-4.96	-6.27	-3.65	<0.05	92.90	380.47	<0.001	0.0018
Orthoptera	FCR	3	10	1.63	0.50	2.75	<0.05	83.49	54.51	<0.001	0.0023
Black soldier fly		23	83	1.82	1.31	2.32	<0.05	82.31	463.61	<0.001	<0.0001
Coleoptera		17	54	4.76	3.91	5.61	<0.05	92.61	717.35	<0.001	<0.0001
Silkworm		4	13	-1.00	-1.80	-0.20	<0.05	91.15	135.55	<0.001	0.1671
Housefly		3	16	3.21	2.14	4.27	<0.05	69.39	49.00	<0.001	<0.0001

95% CI = 95% confidence interval; n = number of studies; k = number of comparisons (treatment vs. control); P(effect) = p-value for the pooled effect size; I^2 = heterogeneity statistic; Q = Cochran's Q statistic; p(Q) = p-value for heterogeneity test; Egger's test p = p-value for publication bias test.

Table S3. Hedges' *g* effect size summary for SGR, WGR and FCR of aquatic species fed dietary black soldier fly (*Hermetia illucens*) meal

Host species	**indicator**	**n**	**k**	Hedges'g	95%Cl(Lower)	95%Cl(Upper)	*p(effect)*	I^2*(%)*
LitoPenaeus vannamei	SGR	12	39	0.28	-0.16	0.73	>0.05	28.84
Procambarus clarkii		1	5	-0.02	-0.14	0.11	>0.05	0.00
Panulirus ornatus		3	11	-0.03	-0.25	0.19	>0.05	5.17
Macrobrachium rosenbergii		2	7	-1.9	-3.32	-0.47	<0.05	58.27
Cherax spp. / Parastacidae		6	14	-0.89	-1.74	-0.04	<0.05	46.88
Scylla paramamosain		1	3	0.33	0.23	0.44	<0.05	0.09
Pelodiscus sinensis		2	8	-0.44	-0.76	-0.12	<0.05	0.80
Eriocheir sinensis		1	5	-1.39	-3.59	0.81	>0.05	41.47
Cherax quadricarinatus	WGR	2	7	-1.82	-3.35	-0.29	<0.05	55.43
Macrobrachium rosenbergii		1	5	-2.65	-4.61	-0.68	<0.05	43.48
LitoPenaeus vannamei		10	36	-4.23	-5.03	-3.42	<0.05	96.08
Scylla paramamosain		1	3	0.36	0.26	0.47	<0.05	0.13
Pelodiscus sinensis		2	8	-0.48	-0.77	-0.20	<0.05	2.17
Eriocheir sinensis		2	9	-2.02	-3.78	-0.26	<0.05	63.88
Cherax cainii		2	3	0.22	-0.14	0.57	>0.05	0.00
Lithobates catesbeianus		1	5	0.01	-0.19	0.21	>0.05	0.00
Procambarus clarkii		1	5	-0.01	-0.14	0.11	>0.05	0.00
Macrobrachium rosenbergii	FCR	1	5	6.53	3.95	9.11	<0.05	72.91
Lithobates catesbeianus		1	5	-0.07	-0.29	0.15	>0.05	0.21
LitoPenaeus vannamei		14	41	0.66	0.03	1.30	<0.05	85.39
Cherax spp. / Parastacidae		2	6	0.62	-1.17	2.40	>0.05	35.24
Procambarus clarkii		2	6	0.48	0.05	0.91	<0.05	43.05
Pelodiscus sinensis		2	8	0.63	0.29	0.97	<0.05	0.00
Eriocheir sinensis		1	4	3.83	0.60	7.06	<0.05	65.10

95% CI = 95% confidence interval; n = number of studies; k = number of comparisons (treatment vs. control); p(effect) = p-value for the pooled effect size; I^2 = heterogeneity statistic.

Table S4. Hedges' *g* effect size summary for SGR, WGR and FCR of aquatic species fed dietary Coleoptera (primarily *Tenebrio molitor*) meal

Host species	**indicator**	**n**	**k**	Hedges'g	95%Cl(Lower)	95%Cl(Upper)	*p(effect)*	*I^2(%)*
LitoPenaeus vannamei	SGR	9	32	-2.28	-3.08	-1.47	<0.05	96.93
Cherax quadricarinatus		1	4	-0.54	-2.76	1.69	>0.05	10.83
Procambarus clarkii		3	6	0.44	-0.18	1.06	>0.05	43.77
Lithobates catesbeianus	WGR	1	4	-0.17	-0.78	0.43	>0.05	4.95
LitoPenaeus vannamei		8	24	-9.60	-11.10	-8.11	<0.05	94.44
Procambarus clarkii		1	3	0.70	-0.92	2.33	>0.05	19.67
Eriocheir sinensis		1	4	-0.68	-3.39	2.03	>0.05	15.66
Cherax quadricarinatus		1	4	-0.52	-1.42	0.39	>0.05	6.67
Cherax quadricarinatus	FCR	1	4	2.93	-0.22	6.07	>0.05	0.00
LitoPenaeus vannamei		12	41	5.97	4.94	7.00	<0.05	93.70
Procambarus clarkii		2	3	-0.12	-0.63	0.39	>0.05	0.00
Eriocheir sinensis		1	4	1.18	-0.54	2.91	>0.05	39.60

Values inside parentheses indicate 95% confidence interval (CI); n = number of studies; k = number of comparisons (treatment vs. control); p(effect) = p-value for the pooled effect size; I^2 = heterogeneity statistic.

Table S5. Summary of linear meta-regression between replacement level and effect size (Hedges' *g*) for SGR, WGR and FCR across all species.

Insect meal category	**indicator**	**Model**	***n***	***k***	Slope	*P*（slope）	Adjusted R^2
Orthoptera	**SGR**	**Linear**	**4**	**10**	0.092	0.341	0.1134
Hermetia illucens			**28**	**86**	-0.019	0.006	0.0864
Coleoptera			**16**	**44**	-0.161	0.045	0.092
Bombyx mori			**5**	**15**	0.034	0.335	0.0717
Musca domestica			**6**	**15**	-0.035	0.220	0.1133
Orthoptera	**WGR**		**3**	**9**	0.110	0.253	0.1813
Hermetia illucens			**22**	**81**	-0.137	<0.001	0.1688
Coleoptera			**13**	**39**	-0.244	0.003	0.2093
Bombyx mori			**4**	**11**	0.052	0.081	0.3008
Musca domestica			**8**	**28**	-0.063	0.555	0.0135
Orthoptera	**FCR**		**3**	**10**	-0.135	0.235	0.1712
Hermetia illucens			**23**	**83**	0.082	<0.001	0.1632

Coleoptera			**17**	**54**	0.154	0.009	0.1232
Bombyx mori			**4**	**12**	-0.061	0.287	0.1121
Musca domestica			**3**	**16**	0.068	0.053	0.2418

n = number of studies; k = number of comparisons (treatment vs. control); Slope = regression coefficient of replacement level (0–100%) on effect size; P(slope) = p-value for the slope; Adjusted R^2 = adjusted coefficient of determination.

Table S6. Summary of linear meta-regression between replacement level and effect size (Hedges' *g*) for SGR, WGR and FCR in Pacific white shrimp (*LitoPenaeus vannamei*).

Insect meal category	**indicator**	**Model**	**n**	**k**	Slope	P（slope）	Adjusted R^2
Black soldier fly	**SGR**	**Linear**	**13**	**41**	-0.013	0.189	0.0439
Coleoptera			**11**	**34**	-0.217	0.048	0.1164
Housefly			**2**	**7**	-0.045	0.232	0.2698
Silkworm			**3**	**10**	-0.026	0.503	0.058
Orthoptera			**3**	**9**	0.266	0.182	0.239
Black soldier fly	**WGR**		**10**	**36**	-0.283	<0.001	0.3242
Coleoptera			**9**	**27**	-0.272	0.018	0.203
Housefly			**2**	**7**	-0.028	0.138	0.3839
Silkworm			**2**	**8**	-0.001	0.527	0.0631
Orthoptera			**1**	**5**	0.045	0.028	0.8421
Black soldier fly	**FCR**		**12**	**39**	0.144	<0.001	0.3051
Coleoptera			**12**	**41**	0.222	0.006	0.1779

Housefly			**4**	**13**	0.026	0.016	0.4237
Silkworm			**3**	**10**	0.009	0.804	0.0072
Orthoptera			**3**	**10**	-0.132	0.201	0.1712

n = number of studies; *k* = number of comparisons (treatment vs. control); Slope = regression coefficient of replacement level (0–100%) on effect size; *P*(slope) = *p*-value for the slope; Adjusted R^2 = adjusted coefficient of determination.

Supplementary S1 References of the 69 studies included in the meta-analysis.

1. Peh, K.-L., Shapawi, R. and Lim, L.-S., 2021. Black cricket (Gryllus bimaculatus) meal as a protein source in the practical diets for juvenile whiteleg shrimp (*LitoPenaeus vannamei*). Iranian Journal of Fisheries Sciences 20. https://doi.org/10.22092/ijfs.2021.124045
2. Dao, T.T.T. and Fotedar, R., 2024. Can Different Dietary Protein Sources Influence the Survival, Growth, and Physiology of 0+ Marron (*Cherax cainii*) Exposed to Feed Deprivation Animals 14: 3591. https://doi.org/10.3390/ani14243591
3. Pan, H.P., Mo, Z.L., Su, Y.P. and Yang, M.L., 2014. Effects of replacing fish meal with Zophobas morio meal on growth and digestive enzyme activity of Scylla serrata [in Chinese]. Feed Industry 35(6): 47-49. https://doi.org/10.13302/j.cnki.fi.2014.06.011
4. Foysal, M.J., Fotedar, R., Tay, C.-Y. and Gupta, S.K., 2019. Dietary supplementation of black soldier fly (*Hermetia illucens*) meal modulates gut microbiota, innate immune response and health status of marron (*Cherax cainii*, Austin 2002) fed poultry-by-product and fishmeal based diets. PeerJ 7: e6891. https://doi.org/10.7717/peerj.6891
5. Shin, J. and Lee, K.-J., 2021. Digestibility of insect meals for Pacific white shrimp (*LitoPenaeus vannamei*) and their performance for growth, feed utilization and immune responses. PLoS ONE 16: e0260305. https://doi.org/10.1371/journal.pone.0260305
6. Li, X., Chen, Y., Zheng, C., Chi, S., Zhang, S., Tan, B. and Xie, S., 2022. Evaluation of Six Novel Protein Sources on Apparent Digestibility in Pacific White Shrimp, *LitoPenaeus vannamei*. Aquaculture Nutrition 2022: 1-11. https://doi.org/10.1155/2022/8225273
7. Li, M.Y., Lv, W., Zhao, Y., Huang, W., Yuan, Q., Yang, H., Wang, A., Zhou, W. and Li, M.Y., 2024. Effects of Substituting *Tenebrio molitor* and Elodea nuttallii as Feed on Growth, Flesh Quality and Intestinal Microbiota of Red Swamp Crayfish (*Procambarus clarkii*). Foods 13: 2292. https://doi.org/10.3390/foods13142292
8. Motte, C., Rios, A., Lefebvre, T., Do, H., Henry, M. and Jintasataporn, O., 2019. Replacing Fish Meal with Defatted Insect Meal (Yellow Mealworm *Tenebrio molitor*) Improves the Growth and Immunity of Pacific White Shrimp (*LitoPenaeus vannamei*). Animals 9: 258. https://doi.org/10.3390/ani9050258
9. Nunes, A.J.P., Yamamoto, H., Simões, J.P., Pisa, J.L., Miyamoto, N. and Leite, J.S., 2023. The Black Soldier Fly (*Hermetia illucens*) Larvae Meal Can Cost-Effectively Replace Fish Meal in Practical Nursery Diets for Post-Larval *Penaeus vannamei* under High-Density Culture. Fishes 8: 605. https://doi.org/10.3390/fishes8120605
10. Saputra, I. and Fotedar, R., 2024. The effect of defatted black soldier fly meal (*Hermetia illucens*) inclusion in the formulated diet on the growth, gene expression, and histopathology of juvenile lobster (Panulirus ornatus Fabricius, 1798). Aquaculture International 32: 11-29. https://doi.org/10.1007/s10499-023-01151-2
11. Sharifinia, M., Bahmanbeigloo, Z.A., Keshavarzifard, M., Khanjani, M.H., Daliri, M., Koochaknejad, E. and Jasour, M.S., 2023. The effects of replacing fishmeal by mealworm (*Tenebrio molitor*) on digestive enzymes activity and hepatopancreatic biochemical indices of *LitoPenaeus vannamei*. Annals of Animal Science. https://doi.org/10.2478/aoas-2022-0098
12. Richardson, A., Dantas-Lima, J., Lefranc, M. and Walraven, M., 2021. Effect of a Black Soldier Fly Ingredient on the Growth Performance and Disease Resistance of Juvenile Pacific White Shrimp (*LitoPenaeus vannamei*). Animals 11: 1450. https://doi.org/10.3390/ani11051450

13. Shin, J., Jo, S., Ko, D. and Lee, K.-J., 2020. Replacing Fish Meal with Black Soldier Fly Larvae and Mealworm Larvae in Diets for Pacific White Shrimp *LitoPenaeus vannamei*. Korean Journal of Fisheries and Aquatic Sciences 53: 900-908. https://doi.org/10.5657/KFAS.2020.0900

14. Zheng, J.Y., Gan, Z.R., Huang, T.Y., Xiao, Y., Xu, W.Y., Li, X.Q. and Leng, X.J., 2024. Replacement of fish meal with defatted black soldier fly (*Hermetia illucens*) in diet of Pacific white shrimp (*LitoPenaeus vannamei*): growth, flesh quality and transcriptome. Journal of Insects as Food and Feed 11: 873-893. https://doi.org/10.1163/23524588-00001235

15. Zheng, J.Y., Gan, Z.R., Huang, T.Y., Xiao, Y., Xu, W.Y., Li, X.Q. and Leng, X.J., 2023. Influence of replacing fish meal with *Hermetia illucens* and *Tenebrio molitor* on the growth and flesh quality of Pacific white shrimp (*LitoPenaeus vannamei*) and its nutrition improvement strategies [in Chinese]. Master's thesis, Shanghai Ocean University.

16. Zhang, L., Liu, X.Q., Yun, B., Qian, X.Q., Jiang, D.H., Yuan, R.M. and Wang, S., 2024. Effects of black soldier fly larvae powder on growth, immunity, and antioxidant performance of Macrobrachium rosenbergii juveniles [in Chinese]. Feed Research 47(4): 63-67. https://doi.org/10.13557/j.cnki.issn1002-2813.2024.04.012

17. Zhang, W.R., Liu, H.X., Wang, Z., Wang, X.J., Wu, Y.B., Qian, Z.C. and Wang, J., 2023. Effect of defatted black soldier fly larvae meal on nutritional quality of *Scylla paramamosain* [in Chinese]. Feed Research 46(5): 51-55. https://doi.org/10.13557/j.cnki.issn1002-2813.2023.05.011

18. Niu, Q.X., Shao, X.P., Ye, J.Y., Qi, C.L., Wu, C.L., Pang, D., Dong, S.L. and Mo, J.Y., 2023. Effects of defatted black soldier fly larvae meal replacing fish meal on growth performance, liver antioxidant capacity, and intestinal microbiota of juvenile Chinese soft-shelled turtle (*Pelodiscus sinensis*) [in Chinese]. Chinese Journal of Animal Nutrition 35(10): 6650-6664. https://doi.org/10.12418/CJAN2023.607

19. Xu, Z.F. and Li, X.Q., 2023. Mechanism analysis of Chlorella sorokiniana meal and *Tenebrio molitor* meal replacing fish meal on growth and immunity of *LitoPenaeus vannamei* [in Chinese]. Master's thesis, Shanghai Ocean University.

20. Tang, Y., 2019. Effects of replacing fish meal with mealworm meal on growth, body composition, digestive enzyme activity, and liver biochemical indices of bullfrog [in Chinese]. Animal Husbandry and Feed Science 40: 35. https://doi.org/10.12160/j.issn.1672-5190.2019.07.009

21. Jiang, D., Lü, F., Wang, A.M., Hu, Y., Chen, T., Qiao, G., Zhang, M.M., Wang, X.Q. and Huang, J.T., 2017. Effects of silkworm pupae meal replacing fish meal on body index, growth performance, and body composition of juvenile Chinese soft-shelled turtle (*Pelodiscus sinensis*) [in Chinese]. China Feed 2017(7): 26-30. https://doi.org/10.15906/j.cnki.cn11-2975/s.20170705

22. Wang, H., Li, E., Huang, Q., Liu, J., Miao, Y., Wang, X., Qin, C., Qin, J. and Chen, L., 2024. Growth and Hepatopancreas Health of Juvenile Chinese Mitten Crab (*Eriocheir sinensis*) Fed Different Levels of Black Soldier Fly (*Hermetia illucens*) Larvae Meal for Fish Meal Replacement. Aquaculture Nutrition 2024: 6625061. https://doi.org/10.1155/2024/6625061

23. Yang, Q., Fan, R., Ma, Z., Jiang, S., Huang, J., Yang, L., Li, Y., Yang, R., Hu, J., Zhou, S., Su, Q. and Zhou, F.-L., 2023. Effects of replacing dietary fishmeal with zymolytic black soldier fly larvae on the growth performance of the mud crab (*Scylla paramamosain*) larvae. Israeli Journal of Aquaculture - Bamidgeh 75(2). https://doi.org/10.46989/001c.89728

24. De León-Ramírez, J.J., García-Trejo, J.F., Sosa-Ferreyra, C.F., Martínez-Ramos, S.A., Chávez-Jaime, R. and Robles-Bustos, D.A., 2021. Fishmeal replacement using housefly larvae meal as protein ingredient in balanced feeds for bullfrog tadpoles and froglets (*Lithobates catesbeianus*). Landbauforschung - Journal of Sustainable Organic Agricultural Systems 71(1): 23-29.

25. Cao, J., Yan, J., Huang, Y., Wang, G., Zhang, R., Chen, X., Wen, Y. and Zhou, T., 2012. Effects of replacement of fish meal with housefly maggot meal on growth performance, antioxidant and non-specific immune indexes of juvenile *LitoPenaeus vannamei* [in Chinese]. Journal of Fisheries of China 36: 529. https://doi.org/10.3724/SP.J.1231.2012.27621

26. Chen, Y.-K., 2023. Effects of black soldier fly larvae meal on growth, immunity and lipid metabolism of *LitoPenaeus vannamei* [in Chinese]. Acta Hydrobiologica Sinica 47: 269. https://doi.org/10.7541/2023.2022.0201

27. Cheng, D.H. and Xie, Z.G., 2012. Effects of dietary protein level and animal protein sources on the survival and growth of *Procambarus clarkii* [in Chinese]. Journal of Anhui Agricultural Sciences 40(22): 11311-11313. https://doi.org/10.13989/j.cnki.0517-6611.2012.22.140

28. Han, G.M., Zhang, J.H., Wu, L.M., Kou, X.M., Ye, X.M., Qin, B.L., Bi, J.H., Wang, S.H., Xu, R., Ma, L.J., Zhang, C.X. and Yang, T., 2023. Effects of black soldier fly larvae meal replacing fish meal on growth, body composition, and immunity of *Procambarus clarkii* [in Chinese]. Chinese Journal of Animal Nutrition 35(7): 4495-4506. https://doi.org/10.12418/CJAN2023.418

29. Hu, J., Wang, G., Huang, W., Zhao, H., Mo, W. and Huang, Y., 2019. Effects of fish meal replacement by black soldier fly (*Hermetia illucens*) larvae meal on growth performance, body composition, serum biochemical indexes and antioxidant ability of juvenile *LitoPenaeus vannamei* [in Chinese]. Chinese Journal of Animal Nutrition 31: 5292-5300.

30. Hu, S.C., Mai, K.S. and Zhang, C.X., 2017. Effects of defatted silkworm pupae meal replacing fish meal on growth, hepatopancreas histological structure, and molting cycle of *LitoPenaeus vannamei* [in Chinese]. Master's thesis, Jimei University.

31. Huang, W.Q., Wang, Y.G., Zhang, Y., Wu, X.Z., Li, B.F., Shi, L., Zang, M. and Zhou, F.F., 2021. Effects of black soldier fly meal replacing fish meal on survival, growth, and water environment of *Scylla paramamosain* [in Chinese]. Feed Research 44(16): 48-51. https://doi.org/10.13557/j.cnki.issn1002-2813.2021.16.011

32. Cai, Y.W., Leng, X.J. and Xue, M., 2022. Effects of fish meal replacement by three protein sources with different processing methods on physical pellet quality and growth performance of Pacific white shrimp (*LitoPenaeus vannamei*) [in Chinese]. Master's thesis, Shanghai Ocean University.

33. Zarantoniello, M., Chemello, G., Ratti, S., Pulido-Rodríguez, L.F., Daniso, E., Freddi, L., Salinetti, P., Nartea, A., Bruni, L., Parisi, G., Riolo, P. and Olivotto, I., 2023. Growth and Welfare Status of Giant Freshwater Prawn (Macrobrachium rosenbergii) Post-Larvae Reared in Aquaponic Systems and Fed Diets including Enriched Black Soldier Fly (*Hermetia illucens*) Prepupae Meal. Animals 13: 715. https://doi.org/10.3390/ani13040715

34. Zhang, H.Q., Zhou, F., Wang, W.P., Xu, X.J., Zhang, J.R. and He, Z.Y., 2013. Effects of housefly maggot meal instead of fish meal on growth performance, textural mechanical properties, and serum parameters in *Pelodiscus sinensis* Japanese strain [in Chinese]. Acta Agriculturae Zhejiangensis 25(2): 225-229. https://doi.org/10.3969/j.issn.1004-1524.2013.02.04

35. Chen, N.S., Wei, T.T. and Liao, Y.Z., 2007. Effects of housefly larva meal and β-glucan on growth and immunity of *LitoPenaeus vannamei* [in Chinese]. Journal of Fisheries of China 31(6): 771-777.

36. Zhao, H.M., Fan, J.H., Zhong, R.P., Zhou, H.P., Qi, Y.X. and Li, G.H., 2017. Effects of dried housefly maggot meal on growth performance of juvenile *Pelodiscus sinensis* [in Chinese]. Heilongjiang Animal Science and Veterinary Medicine 2017(12): 186-187. https://doi.org/10.13881/j.cnki.hljxmsy.2017.2109

37. Mauro-Félix, A.K., Molinero, M., del-Rio-Zaragoza, O.B., Tinajero-Chavez, A., Arambul-Muñoz, E., Peña-Marín, E.S. and Viana, M.T., 2025. Effect of cricket meal and acid cricket silage from Acheta domesticus in diets and as a substitute for fish meal and fish oil on the productive performance of *LitoPenaeus vannamei*. Aquaculture International 33: 105. https://doi.org/10.1007/s10499-024-01777-w

38. Rajalakshmi, K., Felix, N., Ranjan, A., Arumugam, U., Nazir, M.I. and Sathishkumar, G., 2025. Effects of diets formulated with different combinations of novel feed ingredients on growth performance, apparent digestibility, digestive enzymes and gene expression activities of Pacific white shrimp, *Penaeus vannamei*. Aquaculture International 33: 120. https://doi.org/10.1007/s10499-024-01803-x

39. Foysal, M.J., Fotedar, R., Siddik, M.A.B., Chaklader, M.R. and Tay, A., 2021. Lactobacillus plantarum in black soldier fly (*Hermetia illucens*) meal modulates gut health and immunity of freshwater crayfish (*Cherax cainii*). Fish & Shellfish Immunology 108: 42-52. https://doi.org/10.1016/j.fsi.2020.11.020

40. He, Y., Liu, X., Zhang, N., Wang, S., Wang, A., Zuo, R. and Jiang, Y., 2022. Replacement of Commercial Feed with Fresh Black Soldier Fly (*Hermetia illucens*) Larvae in Pacific White Shrimp (*LitoPenaeus vannamei*). Aquaculture Nutrition 2022: 1-8. https://doi.org/10.1155/2022/9130400

41. Rahimnejad, S., Hu, S., Song, K., Wang, L., Lu, K., Wu, R. and Zhang, C., 2019. Replacement of fish meal with defatted silkworm (Bombyx mori L.) pupae meal in diets for Pacific white shrimp (*LitoPenaeus vannamei*). Aquaculture 510: 150-159. https://doi.org/10.1016/j.aquaculture.2019.05.054

42. Langer, S., Bakhtiyar, Y. and Lakhnotra, R., 2011. Replacement of fishmeal with locally available ingredients in diet composition of Macrobrachium dayanum. African Journal of Agricultural Research 6(5): 1080-1084.

43. Sathishkumar, G., Felix, N., Ranjan, A., Nazir, M.I., Prabu, E. and Manikandan, K., 2023. Substituting dietary fishmeal with silkworm pupae meal in diets of Pacific white shrimp (*Penaeus vannamei*): Effects on growth performance, nutrient utilisation, whole-body composition and digestive enzyme activities. Indian Journal of Fisheries 70. https://doi.org/10.21077/ijf.2023.70.3.133916-11

44. Yao, W., Zhang, C., Zhang, S., Hua, G., Zhao, S., Shuang, H., Sun, N., Sun, Y., Poolsawat, L., Wang, J. and Wang, Q., 2024. The Potential of Defatted Yellow Mealworm (*Tenebrio molitor*) Meal as an Alternative Protein Source for Juvenile Chinese Mitten Crab (*Eriocheir sinensis*). Aquaculture Nutrition 2024: 8782924. https://doi.org/10.1155/2024/8782924

45. Alvanou, M.V., Kyriakoudi, A., Makri, V., Lattos, A., Feidantsis, K., Papadopoulos, D.K., Georgoulis, I., Apostolidis, A.P., Michaelidis, B., Mourtzinos, I., Asimaki, A., Karapanagiotidis, I.T. and Giantsis, I.A., 2023. Effects of dietary substitution of fishmeal by black soldier fly (*Hermetia illucens*) meal on growth performance, whole-body chemical composition, and fatty acid profile of Pontastacus leptodactylus juveniles. Frontiers in Physiology 14: 1156394. https://doi.org/10.3389/fphys.2023.1156394

46. Chen, Y., Chi, S., Zhang, S., Dong, X., Yang, Q., Liu, H., Tan, B. and Xie, S., 2022. Effect of black soldier fly (*Hermetia illucens*) larvae meal on lipid and glucose metabolism of Pacific white shrimp *LitoPenaeus vannamei*. British Journal of Nutrition 128: 1674-1688. https://doi.org/10.1017/S0007114521004670

47. Chu, J.-H. and Huang, T.-W., 2024. Evaluation of Black Soldier Fly Larvae Meal on Growth, Body Composition, Immune Responses, and Antioxidant Capacity of Redclaw Crayfish (*Cherax quadricarinatus*) Juveniles. Animals 14: 404. https://doi.org/10.3390/ani14030404

48. Wei, H., Tan, B., Yang, Q., Mao, M., Lin, Y. and Chi, S., 2023. Growth, nonspecific immunity, intestinal flora, hepatopancreas, and intestinal histological results for *LitoPenaeus vannamei* fed with diets supplemented with different animal by-products. Aquaculture Reports 29: 101521. https://doi.org/10.1016/j.aqrep.2023.101521

49. Wang, T., Wang, X., Shehata, A.I., Wang, R., Yang, H., Wang, Y., Wang, J. and Zhang, Z., 2022. Growth performance, physiological and antioxidant capacity responses to dietary fish meal replacement with insect meals for aquaculture: A case study in red claw crayfish (*Cherax quadricarinatus*). Aquaculture Research 53: 3853-3864. https://doi.org/10.1111/are.15892

50. Wang, G., Peng, K., Hu, J., Mo, W., Wei, Z. and Huang, Y., 2021. Evaluation of defatted *Hermetia illucens* larvae meal for *LitoPenaeus vannamei*: effects on growth performance, nutrition retention, antioxidant and immune response, digestive enzyme activity and hepatic morphology. Aquaculture Nutrition 27: 986-997. https://doi.org/10.1111/anu.13240

51. Mazlum, Y., Turan, F. and Bircan Yıldırım, Y., 2021. Evaluation of mealworms (*Tenebrio molitor*) meal as an alternative protein source for narrow-clawed crayfish (Pontastacus leptodactylus) juveniles. Aquaculture Research 52: 4145-4153. https://doi.org/10.1111/are.15253

52. Panini, R.L., Pinto, S.S., Nóbrega, R.O., Vieira, F.N., Fracalossi, D.M., Samuels, R.I., Prudêncio, E.S., Silva, C.P. and Amboni, R.D.M.C., 2017b. Effects of dietary replacement of fishmeal by mealworm meal on muscle quality of farmed shrimp *LitoPenaeus vannamei*. Food Research International 102: 445-450. https://doi.org/10.1016/j.foodres.2017.09.017

53. Zheng, Y., Hou, C., Chen, J., Wang, H., Yuan, H., Hu, N., Shi, L. and Zhang, S., 2023. Integrating microbiome and transcriptome analyses to understand the effect of replacing fishmeal with *Tenebrio molitor* meal in Pacific white shrimp (*LitoPenaeus vannamei*) diets. Aquaculture 575: 739818. https://doi.org/10.1016/j.aquaculture.2023.739818

54. Cai, Y., Huang, H., Yao, W., Yang, H., Xue, M., Li, X. and Leng, X., 2022. Effects of fish meal replacement by three protein sources on physical pellet quality and growth performance of Pacific white shrimp (*LitoPenaeus vannamei*). Aquaculture Reports 25: 101210. https://doi.org/10.1016/j.aqrep.2022.101210

55. Shang, R., Man, L., Wang, G., Li, M., Liu, C. and Li, L., 2022. Influences of Partial Substitution of Fish Meal with Defatted Black Soldier Fly (*Hermetia illucens*) Larvae Meal in Diets on Growth Performance, Biochemical Parameters, and Body Composition of Juvenile Chinese Soft-Shelled Turtles (*Pelodiscus sinensis*). Aquaculture Nutrition 2022: 1-10. https://doi.org/10.1155/2022/4278137

56. Chang, T., Lin, H., Han, F., Xu, C. and Li, E., 2025. Impact of defatted black soldier fly (*Hermetia illucens*) larvae meal on health, muscle texture, and intestinal microbiota in Pacific white shrimp (*Penaeus vannamei*). Aquaculture 596: 741755. https://doi.org/10.1016/j.aquaculture.2024.741755

57. Panini, R.L., Freitas, L.E.L., Guimarães, A.M., Rios, C., Da Silva, M.F.O., Vieira, F.N., Fracalossi, D.M., Samuels, R.I., Prudêncio, E.S., Silva, C.P. and Amboni, R.D.M.C., 2017a. Potential use of mealworms as an alternative protein source for Pacific white shrimp: Digestibility and performance. Aquaculture 473: 115-120. https://doi.org/10.1016/j.aquaculture.2017.02.008

58. Mastoraki, M., Vlahos, N., Patsea, E., Chatzifotis, S., Mente, E. and Antonopoulou, E., 2020. The effect of insect meal as a feed ingredient on survival, growth, and metabolic and antioxidant response of juvenile prawn Palaemon adspersus (Rathke, 1837). Aquaculture Research 51: 3551-3562. https://doi.org/10.1111/are.14692

59. Keetanon, A., Chuchird, N., Phansawat, P., Kitsanayanyong, L., Chou, C.-C., Verstraete, P., Ménard, R., Richards, C.S., Ducharne, F. and Rairat, T., 2024. Effects of black soldier fly larval meal on the growth performance, survival, immune responses, and resistance to Vibrio parahaemolyticus infection of Pacific white shrimp (*LitoPenaeus vannamei*). Aquaculture International 32: 2233-2248. https://doi.org/10.1007/s10499-023-01267-5

60. Usman, U., Fahrur, M., Kamaruddin, K., Asaad, A.I.J. and Fahmi, M.R., 2021. The utilization of black soldier fly larvae meal as a substitution of fish meal in diet for white shrimp, *LitoPenaeus vannamei*, grow-out. IOP Conference Series: Earth and Environmental Science 860: 012023. https://doi.org/10.1088/1755-1315/860/1/012023

61. Saputra, I., Lee, Y.N. and Fotedar, R., 2024. The Effect of Supplementation of Fish Protein Hydrolysate to the BSF-Based Aquafeed on the Growth, Survival, Fatty Acids, and Histopathology of Juvenile Lobster (Panulirus ornatus). Aquaculture Nutrition 2024: 8579991. https://doi.org/10.1155/2024/8579991

62. Lin, H., Liang, X., Han, F., Luo, X. and Li, E., 2023. Growth, Biochemical Characteristics, Flesh Quality, and Gut Microbiota of the Pacific White Shrimp (*Penaeus vannamei*) Fed a Defatted Superworm (Zophobas atratus) Larvae Meal. Aquaculture Nutrition 2023: 1-25. https://doi.org/10.1155/2023/8627246

63. Choi, I.H., Kim, J.M., Kim, N.J., Kim, J.D., Park, C., Park, J.-H. and Chung, T.H., 2018. Replacing fish meal by mealworm (*Tenebrio molitor*) on the growth performance and immunologic responses of white shrimp (*LitoPenaeus vannamei*). Acta Scientiarum Animal Sciences 40: 39077. https://doi.org/10.4025/actascianimsci.v40i1.39077

64. Cummins, V.C., Rawles, S.D., Thompson, K.R., Velasquez, A., Kobayashi, Y., Hager, J. and Webster, C.D., 2017. Evaluation of black soldier fly (*Hermetia illucens*) larvae meal as partial or total replacement of marine fish meal in practical diets for Pacific white shrimp (*LitoPenaeus vannamei*). Aquaculture 473: 337-344. https://doi.org/10.1016/j.aquaculture.2017.02.022

65. Thirumurugan, R. and Subramanian, P., 2004. Growth of juvenile freshwater prawn Macrobrachium malcolmsonii fed with isonitrogenous diets containing different biowastes. Journal of Food Science and Technology (Mysore) 41(1): 95-99.

66. Yao, W., Zhang, C., Mao, H., Hua, G., Liu, Q., Zhao, S., Shuang, H., Poolsawat, L., Yuan, S., Wang, J. and Wang, Q., 2024. Effects of dietary defatted black soldier fly (*Hermetia illucens*) larvae meal substituting fish meal on growth, antioxidative capacity, immunity, intestinal histology and microbiota of juvenile Chinese mitten crab (*Eriocheir sinensis*). Aquaculture Reports 38: 102302. https://doi.org/10.1016/j.aqrep.2024.102302

67. Li, X., Rahimnejad, S., Wang, L., Lu, K., Song, K. and Zhang, C., 2019. Substituting fish meal with housefly (*Musca domestica*) maggot meal in diets for bullfrog Rana (*Lithobates*) catesbeiana: Effects on growth, digestive enzymes activity, antioxidant capacity and gut health. Aquaculture 499: 295-305. https://doi.org/10.1016/j.aquaculture.2018.09.053

68. Zhou, L., Chen, K., Song, K., Lu, K., Li, X., Wang, L. and Zhang, C., 2025. Black soldier fly (*Hermetia illucens Linnaeus*) larvae meal - a promising protein source in bullfrog (*Aquarana catesbeiana*) feed. Aquaculture 608: 742687. https://doi.org/10.1016/j.aquaculture.2025.742687

69. Tang, J., Dai, Y., Liang, X., Zhang, Y., Huang, F., Lou, B. and Guo, S., 2025. Evaluation of common housefly *Musca domestica* maggot meal as partial substitution of fish meal and fish oil in Chinese mitten crab *Eriocheir sinensis* diets. Aquaculture Reports 41: 102709. https://doi.org/10.1016/j.aqrep.2025.102709
