# Supplementary figures and images for: Effects of Fishmeal Replacement with Insect Meals on Growth Performance in Non-Fish Aquatic Animals: A Meta-Analysis
Source: Insects. 2026 Jul 6;17(7):699. doi: 10.3390/insects17070699 (PMC13409791; doi:10.3390/insects17070699)

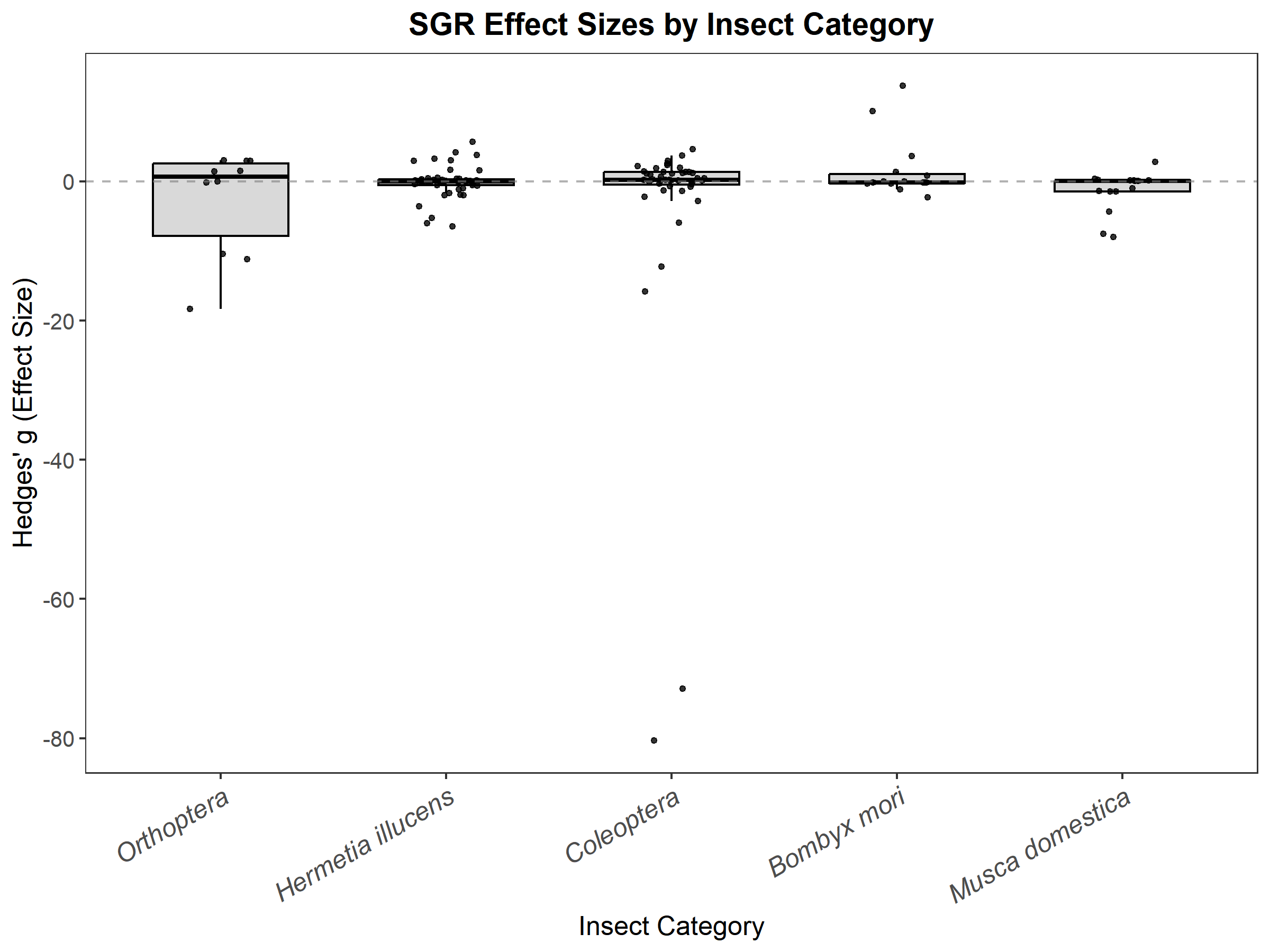

Supplement: Supplementary file 1 [file insects-17-00699-s001.zip › Figure_S1.png]

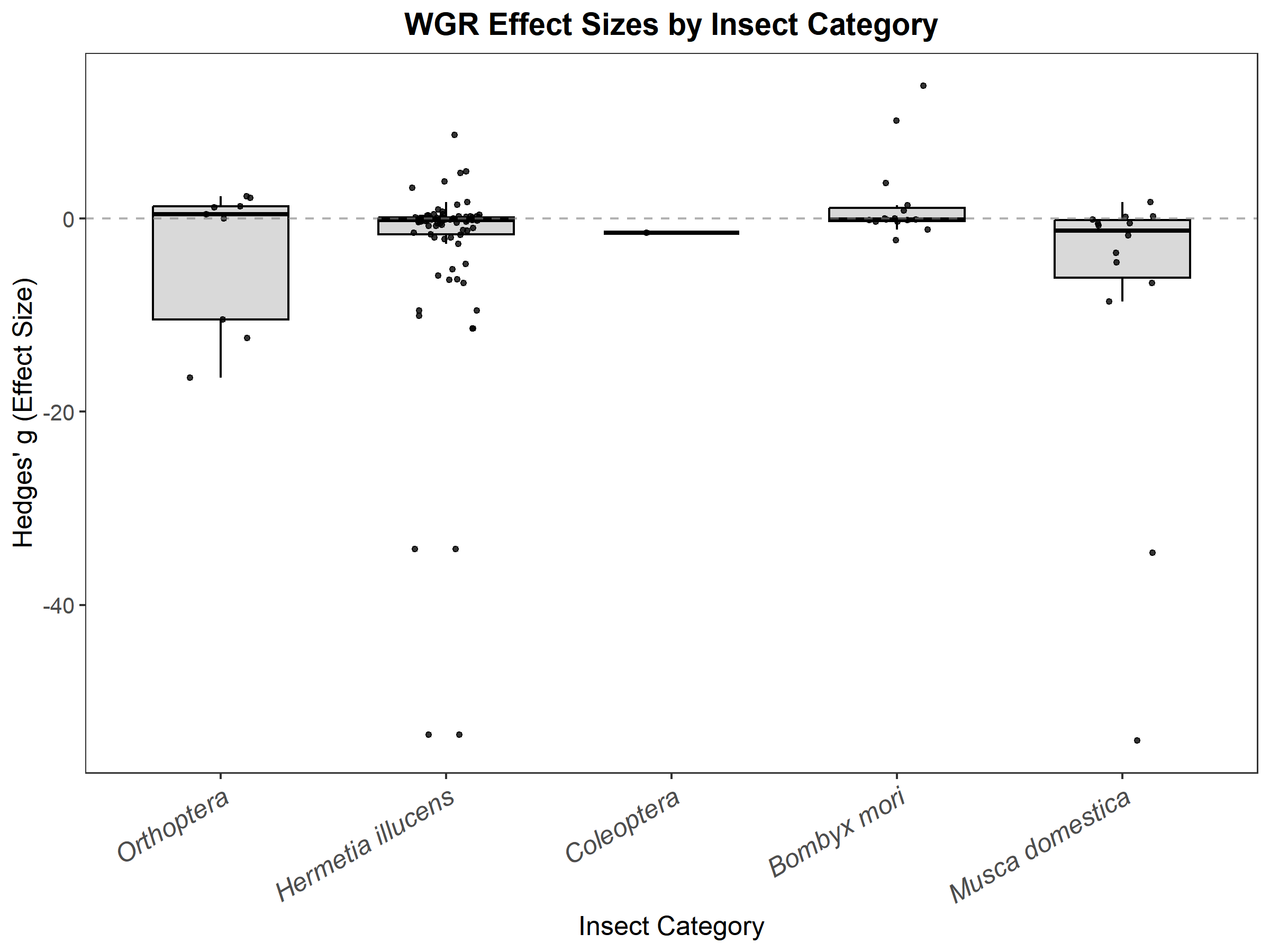

Supplement: Supplementary file 1 [file insects-17-00699-s001.zip › Figure_S2.png]

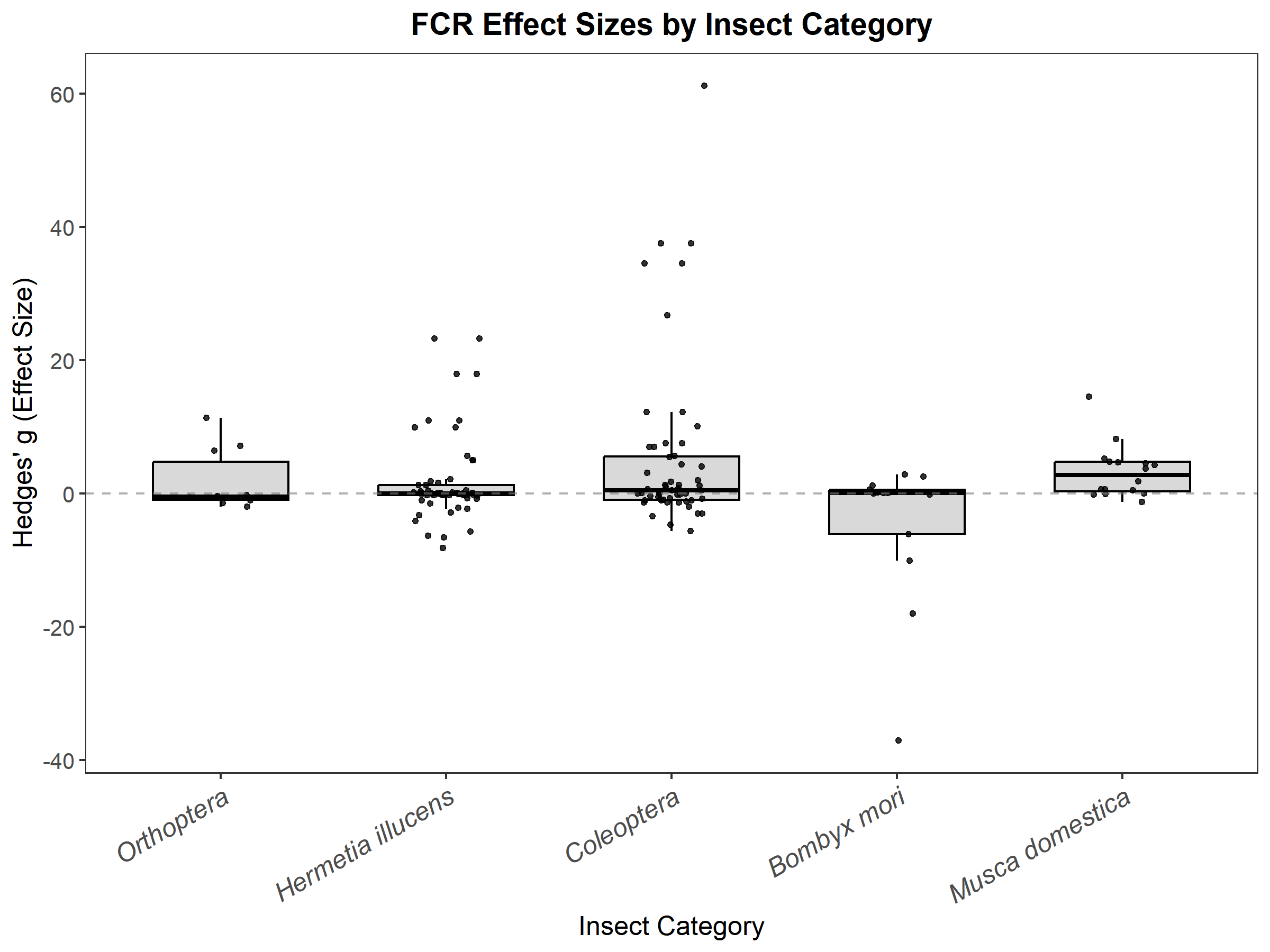

Supplement: Supplementary file 1 [file insects-17-00699-s001.zip › Figure_S3.png]
